# Localized force application reveals mechanically sensitive domains of Piezo1

**DOI:** 10.1038/ncomms12939

**Published:** 2016-10-03

**Authors:** Jason Wu, Raman Goyal, Jörg Grandl

**Affiliations:** 1 Duke University Medical Center, Department of Neurobiology, Durham, North Carolina 27710, USA

## Abstract

Piezos are mechanically activated ion channels that function as sensors of touch and pressure in various cell types. However, the precise mechanism and structures mediating mechanical activation and subsequent inactivation have not yet been identified. Here we use magnetic nanoparticles as localized transducers of mechanical force in combination with pressure-clamp electrophysiology to identify mechanically sensitive domains important for activation and inactivation.

## Abstract (web summary)

Piezo ion channels transduce mechanical forces into biological signals. Here, the authors use magnetic tweezers to pull specific regions of Piezo1, identifying mechanically sensitive domains that are essential for channel activation and inactivation.

## Graphical abstract

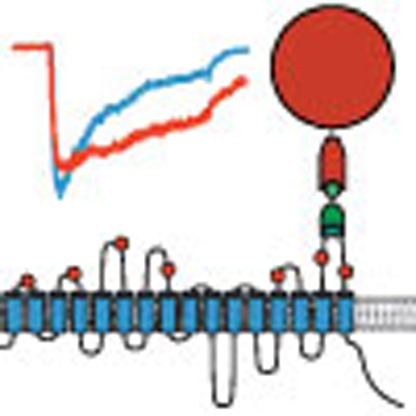

Piezos are large (∼2,500 aa.) proteins with 14–38 transmembrane domains that form mechanically activated ion channels^1^,^2^,^3^,^4^. They have been implicated in several biological processes involving mechanical sensing such as the sense of touch, proprioception and cardiovascular development^5^,^6^,^7^,^8^,^9^,^10^.

Recent studies have demonstrated that lateral membrane tension is the physical stimulus that activates Piezo1, suggesting hydrophobic mismatch between the membrane bilayer and transmembrane domains as a possible mechanism of mechanical sensing^11^,^12^. However, Piezos are unrelated to other known ion channel families, and therefore, the precise mechanism that transduces mechanical force into pore opening (activation) and subsequently leads to pore closing (inactivation) is unknown.

The macroscopic organization of Piezo1 has been revealed by cryo-electron microscopy, and a smaller (∼230 aa.) extracellular domain of the *C. elegans* Piezo orthologue was resolved at the atomic level^4^,^13^. The C-terminal region contains the pore domain and is also highlighted by several disease-related single-point mutations that cause a slowing of inactivation kinetics^2^,^14^,^15^,^16^. However, further detailed links between structural domains and distinct modalities of channel function such as activation and inactivation have remained unresolved. Existing thermodynamic models of mechanical gating simplify channel structure to be homogenous and elastic and are thus limited in revealing structural features^17^. Here we aimed to advance our understanding of this concept, hypothesizing that specific structures within Piezos are highly sensitive to localized application of force, whereas others are less sensitive in comparison. We further reasoned that mechanical perturbation of such domains may induce changes in channel function.

In this study, we introduce a method by which localized force is applied through magnetic nanoparticles to distinct domains of Piezo1. We identify two domains that are mechanically sensitive and affect pressure-dependent channel activation and inactivation.

## Results

### Localized force application by magnetic nanoparticles

**Figure 1:**
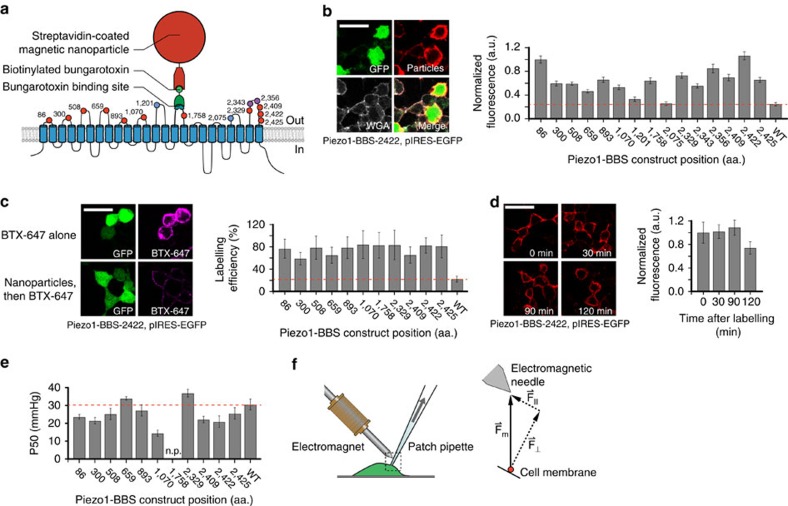
Localized force application by nanoparticle labelling and magnetic field generation. (**a**) Piezo1 transmembrane topology with aa. locations of BBS insertions (red, labelled and functional; blue, non-labelled; magenta, non-functional) and schematic of bead labelling strategy. (**b**) Representative images of HEK293T cells expressing Piezo1-BBS-2422-pIRES-EGFP construct, live-labelled with streptavidin-coated nanoparticles, immunostained against streptavidin, and labelled with WGA to confirm membrane localization (green, GFP; red, anti-streptavidin; grey, WGA). Mean fluorescence intensity normalized to BBS-86 (a.u.) of nanoparticle labelling along the cell membrane for all constructs compared with wild-type Piezo1 (WT, red line) (*n*=10 cells per transfection, 2–5 transfections; *P*<0.0001 for all constructs except BBS-1201 and BBS-2075 (*P*>0.01), one-way ANOVA and NP multiple comparison). (**c**) Representative images of HEK293T cells expressing BBS-2422, live-labelled with either bungarotoxin (BTX)-Alexa Fluor 647 alone or first with nanoparticles, then followed by BTX-647 (green, GFP; magenta, BTX-647). Quantified labelling efficiency for all Piezo1-BBS constructs in comparison to WT (red line) (*n*=12 cells each, 1 transfection; *P*<0.0001, one-way ANOVA and NP multiple comparison). (**d**) Representative fluorescent images of HEK293T cells transfected with BBS-2422 as a function of time after surface labelling (red, anti-streptavidin) and mean membrane fluorescence intensity normalized against *t*=0 time point plotted versus time. (*n*=10 cells per transfection, 1–2 transfections; no significance between any time points, *P*>0.01, one-way ANOVA and Tukey's multiple comparison). (**e**) Average pressure of half-maximal activation (*P*_50_) for each construct (*n*=8–21 cells, 3–7 transfections) in comparison to wild-type Piezo1 (red line) (*P*<0.0001 for BBS-1070, one-way ANOVA and NP multiple comparison). Construct BBS-1758 did not reach a plateau current (n.p.), and a *P*_50_ value was not measured. (**f**) Diagram of patch-clamp pipette and electromagnetic needle and corresponding force diagram on nanoparticle (**F**_m_, magnetic force vector; **F**_⊥_, force vector normal to patch membrane; **F**_||_, force vector parallel to patch membrane). Error bars are s.e.m. for all panels. Scale bar, 30 μm.

To probe mechanical sensitivity of Piezo1 ion channels with sub-molecular resolution, we generated a highly localized pulling force to specific domains of Piezo1 by attaching superparamagnetic nanoparticles and exposing the complex to a magnetic field while measuring channel activity electrophysiologically. Specifically, we engineered constructs of Piezo1-IRES-EGFP that each contained a 13 amino-acid (aa.) bungarotoxin binding sequence (BBS) within a predicted extracellular domain, further referred to as Piezo1-BBS^18^. We first treated HEK293T cells expressing Piezo1-BBS constructs with biotinylated bungarotoxin, which binds to the BBS with high affinity (*K*_d_∼15 nM)^19^. Next, we applied 75 nm diameter streptavidin-coated nanoparticles to the cells, which in turn bind the biotinylated bungarotoxin (*K*_d_∼0.01 pM), linking the targeted domain to the nanoparticle (Fig. 1a). We reasoned that due to the comparatively large size, each Piezo1 channel can accommodate at most one single nanoparticle (Supplementary Fig. 1).

To probe the specificity of nanoparticle labelling, we immunostained nanoparticles bound to cells transfected with Piezo1-BBS constructs or wild-type Piezo1, which does not contain any BBS, and compared their near-membrane fluorescence. All but two Piezo1-BBS constructs (those with BBS tags inserted at residue positions 1,201 and 2,075 (BBS-1201 and BBS-2075)) exhibited a fluorescence intensity that was at least two times higher as compared to the levels present on wild-type Piezo1 expressing cells and were used for further experiments (Fig. 1b; Supplementary Fig. 2). Then, to probe the efficiency of nanoparticle labelling, we labelled unoccupied binding sites of all Piezo1-BBS constructs with a fluorescently conjugated bungarotoxin either directly or after the binding of nanoparticles. We observed for all constructs that prior nanoparticle labelling reduced fluorescence by 60–80% (Fig. 1c; Supplementary Fig. 3a,b). Finally, to probe for possible nanoparticle dissociation or internalization, we immunostained nanoparticle-labelled cells transfected with one representative construct (BBS-2422) at various time points after labelling. We observed consistent fluorescence intensity (1.04±0.08 a.u.) along the membrane over a time period of at least 1.5 h (Fig. 1d). We therefore concluded that labelling of Piezo1-BBS constructs with magnetic nanoparticles was overall sufficiently specific, efficient and stable to be used as localized force transducers.

We next probed by pressure-clamp electrophysiology if Piezo1-BBS constructs retained normal mechanical sensitivity, peak current amplitudes and inactivation kinetics as compared with wild-type Piezo1 (Fig. 1e; Supplementary Fig. 4a,b). The majority of the constructs retained channel properties similar to wild-type Piezo1. Only two constructs (BBS-2343 and BBS-2356 within one domain were non-functional, and for three other domains, we only obtained constructs with attenuated pressure sensitivity (*P*_50_) (BBS-1070 and BBS-1758) or altered inactivation kinetics (BBS-1070 and BBS-2329). We accommodated the rightward shift in *P*_50_ for BBS-1758 with a higher ranged pressure-step protocol (−50 to −120 mm Hg). We decided to study these constructs despite their altered function, because we reasoned that they might still be informative if external pulling force further alters channel function. Altogether, we created eleven functional and accessible constructs, covering eight of the nine individual extracellular loops that were previously identified by affinity-tag accessibility experiments^2^.

Finally, we engineered an electromagnetic coil (magnetic field **B**∼40 mT) with an iron-nickel alloy core needle tapered to a <10 μm tip to generate a focused magnetic field gradient and positioned it 54.7±5.5 μm above the tip of the patch pipette (Fig. 1f; Supplementary Fig. 5a,b)^20^. This relatively large distance between the needle and membrane surface results in a magnetic pulling force that is predominantly perpendicular to the membrane plane, with only a small parallel component.

### Magnetic force induces domain-specific loss of inactivation

**Figure 2:**
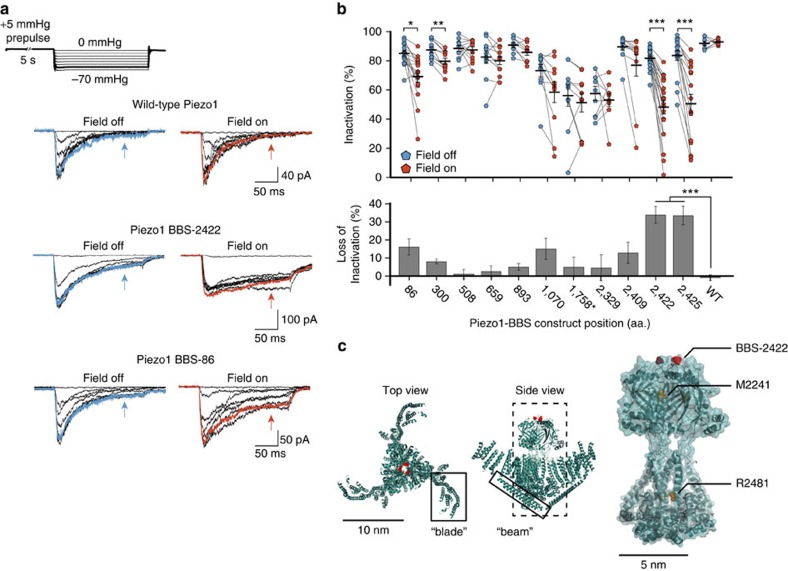
Effect of magnetic pulling force on Piezo1 inactivation. (**a**) Representative current recordings of wild-type Piezo1, construct BBS-2422 and construct BBS-86 on pressure-clamp stimulation from 0 to −70 mm Hg (above) in the absence and presence of a magnetic field. Arrows denote inactivated current after 150 ms. Traces highlighted in bold represent −60 mm Hg step and corresponding current (blue, field off; red, field on). (**b**) Inactivation at −60 mm Hg (or −110 mm Hg for BBS-1758; top panel) for individual experiments and averages of constructs labelled with nanoparticles before (blue) and during (red) application of magnetic force (*n*=8–21 cells, 3–7 transfections; *P<0.01 **P<0.001, ***P<0.0001, paired *t*-test). Average loss of inactivation (bottom panel) for data above (***P<0.0001, one-way ANOVA and Tukey's multiple comparison and NP multiple comparison). Error bars are s.e.m. (**c**) Top and side views (left and middle panels) of *Mm*Piezo1 cryo-EM study (PDB 3JAC) with approximate location of aa. position 2,422 highlighted in red. Previously named ‘blade' and ‘beam' are boxed. Predicted pore region (right) with overlaid electron density, highlighting 2,422 position in red and alignment of human Piezo1 inactivation point mutations in yellow (M2242 and R2481 in mouse).

To measure the effect of magnetic pulling on Piezo1 channel function, we patched HEK293T cells expressing each Piezo1-BBS construct labelled with nanoparticles in the cell-attached configuration. We recorded currents in response to a pressure-step protocol (0 to −70 mm Hg with a +5 mm Hg prepulse) we developed recently that minimizes resting membrane tension, first without a magnetic field and then a second time in the presence of a magnetic field (Fig. 2a)^12^.

As we expected, the magnetic field did not alter channel function when applied to wild-type Piezo1 lacking a BBS, demonstrating that Piezo1 channels have no inherent sensitivity to magnetic fields (Fig. 2a). While some constructs were insensitive to magnetic force when labelled with nanoparticles, we found marked changes in channel function for certain constructs. Most strikingly, construct BBS-2422 revealed a substantial loss of inactivation on magnetic force application, and an identical effect with the BBS at the proximal position in BBS-2425 confirmed region specificity (Fig. 2a,b). A similar, albeit smaller, loss of inactivation was also apparent for BBS-86 and BBS-300 (Fig. 2a,b). Although other mechanically sensitive sites may have been overlooked by our labelling strategy, which sparsely probes an unusually large protein and is restricted to extracellular domains, this result qualitatively supports our hypothesis that distinct sites within the Piezo1 channel are highly sensitive to localized force application, while others are less sensitive.

To quantify the loss of inactivation, we calculated the inactivation (Methods) 150 ms after the peak current evoked by a saturating pressure-step of −60 mm Hg and its change on magnetic force application (Fig. 2b). For example, in BBS-2422 we found that the average inactivation decreased strongly (from 81.7±1.2% (field off) to 48.0±4.8% (field on), *n*=19 cells, *P*<0.0001, paired *t*-test), while in BBS-508 the average inactivation remained unchanged (88.5±2.0% (field off) and 87.5±2.1% (field on), *n*=12 cells, *P*>0.01, paired *t*-test). This result shows quantitatively that inactivation, which is a hallmark of Piezo1 channel function, can be significantly inhibited by localized magnetic force application. Among all Piezo1-BBS constructs, the average loss of inactivation was greatest for BBS-2422 and BBS-2425. In addition, we analysed the change in maximum current amplitude and in pressure sensitivity (*P*_50_) in the presence of a magnetic field for all constructs and found, apart from a small decrease (22.0±5.0%; *P*<0.01, paired *t*-test) in current amplitude for BBS-2422, no significant changes (Supplementary Fig. 6a,b). To test whether the magnetic field could lead to direct channel activation, we analysed the channel open probability (NP_o_) during the +5 mm Hg prepulse period in the presence and absence of the magnetic field. We found NP_o_<1 % for all constructs for both conditions, indicating that the channel was predominantly closed regardless of the presence of a magnetic field (Supplementary Fig. 7).

Altogether, four extracellular sites within two domains emerged from our screen as significantly mechanically sensitive. BBS-86 and BBS-300 are both tagged proximal to the N terminus, and BBS-2422 and BBS-2425 are tagged precisely on top of a large structurally unique extracellular domain located between the two predicted pore helices that likely forms part of the ion permeation pathway (Fig. 2c)^4^,^13^. Due to the robust effect we observed on localized force application at BBS-2422 and the presence of human single-point mutations found within and surrounding this extracellular domain that cause loss of inactivation, we decided to further focus on construct BBS-2422 to better understand how localized force acts on Piezo1 function mechanistically^21^.

### Magnetic force acts directly on channel domains

**Figure 3:**
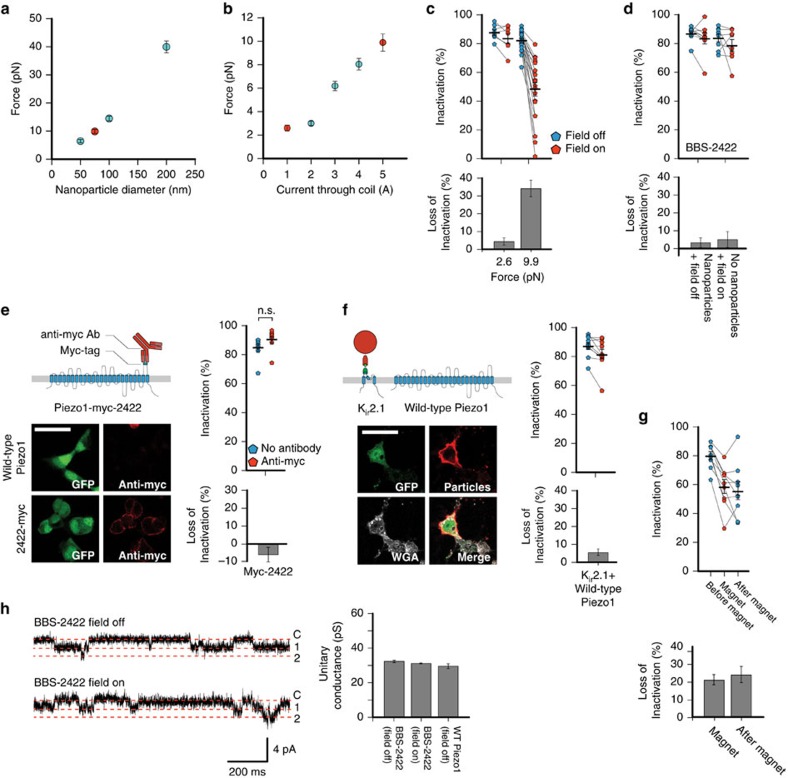
Mechanism of magnetic force on construct BBS-2422. (**a**) Measured force on fluorescently labelled magnetic nanoparticles (red, 75 nm diameter; *n*=15 nanoparticles per size). (**b**) Measured force on fluorescently labelled 75 nm diameter magnetic nanoparticles under varying current supply to electromagnet (red, 1 A and 5 A analyzed in next panel; *n*=15 nanoparticles per condition). (**c**) Inactivation and averages of individual experiments (top) and average differences (bottom) of BBS-2422 with ∼25% (1 A; 2.6±0.2 pN; *n*=8 cells, 4 transfections) and 100% (5 A; 9.9±0.7 pN; *n*=21 cells, 7 transfections) magnetic force with a −60 mmHg pressure step. (**d**) Percent inactivation and averages of individual experiments (top) and average differences (bottom) of construct BBS-2422 labelled with nanoparticles without magnetic field (*n*=8 cells each, 2 transfections) or unlabelled and with magnetic field with a −60 mm Hg pressure step (*n*=9, 2 transfections). (**e**) Schematic of antibody labelling on Piezo1-myc-2422 (pIRES-EGFP) and representative images of HEK293T cells expressing wild-type Piezo1 (pIRES-EGFP) or Piezo1-myc-2422 construct (green, GFP; red, anti-myc). Per cent inactivation and averages of individual experiments (top right) and average differences (bottom right) of myc construct labelled and unlabelled with anti-myc antibody (*n*=8 cells each, 2 transfections; NS, *P*>0.01, unpaired *t*-test). (**f**) Schematic of K_ir_2.1-BBS construct labelled with nanoparticles co-expressed with wild-type Piezo1 (pIRES-EGFP) and representative images of surface labelled HEK293T cells (green, GFP; red, anti-streptavidin; grey, WGA). Inactivation and averages of individual experiments (top right) and average differences (bottom right) for co-expressed K_ir_2.1-BBS and wild-type Piezo1 before and on magnetic force (*n*=10 cells, 2 transfections). Scale bar, 30 μm. (**g**) Inactivation and averages of individual experiments (top) and average differences (bottom) for BBS-2422 recorded first without magnetic field, second with magnetic field, and third again without magnetic field (*n*=9 cells, 5 transfections). (**h**) Representative single-channel recordings for BBS-2422 at resting tension with and without a magnetic field and quantification of single-channel conductance (*n*=9 cells, 4 transfections). Error bars are s.e.m. for all panels.

Measuring the magnitude of the applied pulling force might allow for inferences about its effects on protein stability and function. To calibrate our setup, we labelled the 75 nm magnetic nanoparticles with Alexa Fluor 594 and measured their velocity through glycerol in the magnetic field of our setup. Using Stokes' law, we calculated the drag force on 75 nm nanoparticles to be 9.9±0.7 pN, setting the upper limit of the total force applied to a single Piezo1 channel (Fig. 3a). By performing this measurement on nanoparticles of 50, 100, and 200 nm diameter, we found that force increases with particle size, which is expected given that the magnetic susceptibility is the same for all bead sizes (Ocean NanoTech). In addition, we measured the applied force for varying current amplitudes through the electromagnetic coil (Fig. 3b). Supplying a current of 1 A reduced the force to 2.6±0.2 pN, which is comparable to the force of ambient thermal energy alone (Methods). With this force we were unable to elicit a slowing of inactivation on BBS-2422, supporting the notion that the functional effect we observe is due to a force magnitude that exceeds physiological conditions (Fig. 3c).

Next, we considered the possibility that steric effects of nanoparticle labelling may induce changes in channel activity. We observed for constructs BBS-86, BBS-300, BBS-2422 and BBS-2425 that nanoparticle labelling alone (that is, in the absence of a magnetic field), did not induce a loss of inactivation on two consecutive pressure-step stimulation protocols. Similarly, the presence of a magnetic field alone (that is, without prior nanoparticle labelling) also did not elicit a loss of inactivation (Fig. 3d; Supplementary Fig. 8). To further address the possibility of steric hindrance, we engineered a Piezo1-IRES-EGFP construct with a myc-tag binding sequence at residue 2422 (Piezo1-myc-2422) that we could specifically conjugate with a 10 nm-sized anti-myc antibody (Supplementary Fig. 9). The pressure sensitivity and inactivation (*P*_50_=18.5±2.4 mm Hg, 84.9±2.7%, *n*=8 cells) of this construct were not affected by labelling it with the anti-myc antibody (*P*_50_=19.6±1.7 mm Hg, 90.6±2.5%, *n*=8 cells; *P*>0.01, unpaired *t*-test; Fig. 3e). Altogether, these results support that it is specifically the magnetic force application that affects Piezo function and not steric hindrance.

We next hypothesized that Piezo function may be affected indirectly through pulling force transduced through the membrane bilayer. To test this we tagged the inwardly rectifying potassium K_ir_2.1 channel with a BBS (K_ir_2.1-BBS) on the first extracellular pore loop (S1-pore helix) and co-expressed it with wild-type Piezo1-pIRES-EGFP in HEK293T cells (Fig. 3f; Supplementary Fig. 9). We found that wild-type Piezo1 function (*P*_50_=24.6±2.7 mm Hg, average maximal current=148±44 pA, inactivation=87.4±2.3%, *n*=10 cells) was not affected by the force on K_ir_2.1 channels (*P*_50_=19.7±2.0 mm Hg, average maximal current=154±38 pA, inactivation=81.8%±3.3; *P*>0.01, paired *t*-test). This result, together with the observation that the functional effects we detected were specific to each Piezo1-BBS construct, suggests that the pulling force is acting primarily on the tagged domain and not indirectly though the membrane bilayer.

Interestingly, the loss of inactivation is irreversible on a minute timescale (Fig. 3g). This is comparable to previous reports that a permanent loss of inactivation of wild-type Piezo1 can be mechanically induced^22^. Such an effect suggests that magnetic pulling force on BBS-2422 may be accelerating an existing, but mechanistically unclear, tendency towards channel inactivation.

Structural studies showed that the BBS-2422 site is located near or along the ion permeation pathway^4^. We found that the single-channel conductance *g* of BBS-2422 (*g*=32.5±0.7 pS; *n*=10 cells) remained unchanged by magnetic pulling force (*g*=31.3±0.3 pS; *n*=10 cells; *P*>0.01, paired *t*-test), therefore suggesting that mechanical perturbation of the extracellular domain containing residue 2,422 is directly affecting the mechanism of inactivation independently of ion permeation (Fig. 3h).

### Magnetic force specifically perturbs mechanical gating

**Figure 4:**
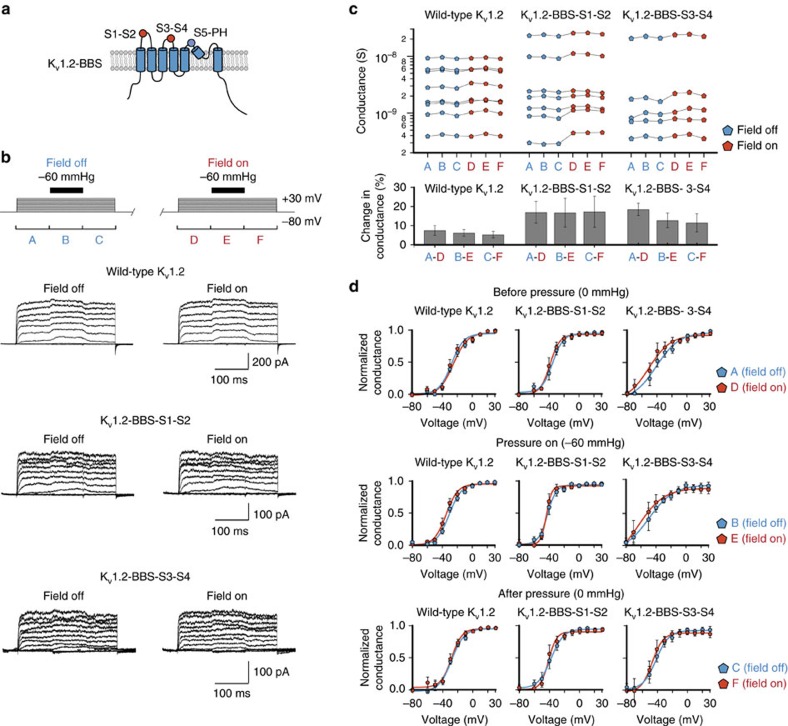
Specificity of magnetic force on mechanical activation. (**a**) K_v_1.2 transmembrane topology with aa. locations of BBS insertions (red, labelled and functional; blue, non-labelled and non-functional). (**b**) Voltage-step and pressure-pulse protocol with representative current traces for wild-type K_v_1.2, K_v_1.2-BBS-S1-S2 and K_v_1.2-BBS-S3-S4. Periods A, C, D, and F mark voltage-stimulation alone, while B and E are marking simultaneous voltage-stimulation and pressure stimulation (field off for A–C, field on for D–F). (**c**) Maximal conductances of individual recordings (*P*>0.01 for comparisons between pairs A–D, B–E and C–F for all constructs, paired *t*-test) and mean percent changes (*n*=5–8 cells, 2 transfections each; *P*>0.01 for comparisons of between corresponding values compared to wild-type K_v_1.2, unpaired *t*-test). (**d**) Normalized average *G*/*V* curves for periods A (field off) versus D (field on), B (field off) versus E (field on), and C (field off) versus F (field on) (*P*>0.01 for comparisons of *V*_1/2_ in each panel, paired *t*-test). Error bars are s.e.m. for all panels.

We next aimed to test whether the observed functional effects from a magnetic pulling force were specific to a mechanism of mechanical activation or rather a nonspecific conformational change. To test this, we inserted a BBS into the extracellular loops of the voltage-activated ion channel K_v_1.2 (K_v_1.2-BBS). While this channel is not known to be mechanically activated, a previous study has shown that mechanosensitivity can be conferred by fusing it with the mechanosensitive ankyrin repeat domain of NOMPC, and another study has shown that activation can be modulated when stimulated with pressure, suggesting that this channel may be a strong candidate for modulation by a magnetic pulling force^23^,^24^. Specifically, a BBS was inserted into the extracellular loops S1-S2 (K_v_1.2-BBS-S1-S2) and S3-S4 (K_v_1.2-BBS-S3-S4) of the voltage-sensing domain, which undergoes a well-characterized conformational change on depolarization leading to channel activation, as well as the S5-pore-helix (K_v_1.2-BBS-S5-PH) of the pore domain (Fig. 4a)^25^. Nanoparticle labelling was specific to constructs K_v_1.2-BBS-S1-S2 and K_v_1.2-BBS-S3-S4 constructs, but failed to label K_v_1.2-BBS-S5-PH (co-transfected with EGFP) (Supplementary Fig. 10). We recorded voltage-activated currents from nanoparticle-labelled K_v_1.2-BBS transfected HEK293 cells in a cell-attached configuration, while additionally applying a transient −60 mm Hg pressure stimulus, analogous to our studies on Piezo1, at each voltage-step (Fig. 4b). As with Piezo1, these recordings were performed first without and then with a magnetic force F=9.9±0.7 pN applied.

In the absence of magnetic force, maximal conductance from wild-type K_v_1.2 expressing cells increased slightly and reversibly on pressure stimulation (5.3±0.6% increase on pressure onset; and 2.6±0.8% decrease on pressure release; *P*<0.0001, *n*=8 cells, paired *t*-test), which recapitulates an inherent mechanical modulation that had been reported before (Fig. 4b)^24^. As expected for a wild-type channel not binding magnetic nanoparticles, magnetic force application did not induce any further statistically significant changes in mean maximal conductance or voltage sensitivity before, during, or after pressure stimulation (change in conductance=7.5±2.6% (before), 6.2±1.8% (during), 5.3±1.7 % (after); for all pairs *P*>0.01, *n*=8 cells, paired *t*-test) (Fig. 4b-d; Supplementary Table 1). Likewise, we did not detect a statistically significant change in conductance, though an increasing trend is observed, or voltage sensitivity on magnetic stimulation at each stage of the stimulation protocol for both constructs targeting the voltage sensor domain: K_v_1.2-BBS-S1-S2 (change in conductance=17.0±5.6% (before), 16.8±7.5% (during), 17.3±8.1% (after); for all pairs *P*>0.01, *n*=7 cells, paired *t*-test) and K_v_1.2-BBS-S3-S4 (change in conductance=18.5±3.2% (before), 12.8±3.8% (during), 11.5±4.7% (after); for all pairs *P*>0.01, *n*=5 cells, paired *t*-test; Fig. 4b-d; Supplementary Table 1). Also, wild-type K_v_1.2 and both K_v_1.2-BBS constructs did not exhibit any inactivation, and again this property was unaffected by magnetic stimulation (Fig. 4b ; Supplementary Table 1). Construct K_v_1.2-BBS-S5-PH, targeting the pore domain, did not exhibit any currents above those measured in non-transfected cells, which was consistent with its lack of nanoparticle labelling, showing that K_v_1.2 did not tolerate insertion of BBS into the very short extracellular pore loop. Together, these results support that our approach specifically identified mechanically sensitive regions of Piezo1.

**Figure 5:**
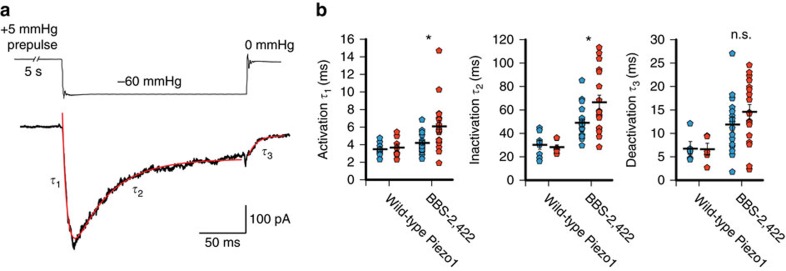
Magnetic force affects mechanical activation. (**a**) Representative current trace of BBS-2422 on stimulation by a −60 mm Hg pressure step and exponential fit functions (red) with time constants of activation (*τ*_1_), inactivation (*τ*_2_), and deactivation (*τ*_3_). (**b**) Time constants and averages of activation, inactivation, and deactivation obtained from individual experiments without magnetic field (blue) and on magnetic field (red) for wild-type Piezo1 (*n*=8 cells, 3 transfections) and construct BBS-2422 (*n*=17 cells, 7 transfections; *P<0.01, n.s., *P*>0.01, paired *t*-test). Error bars are s.e.m. for all panels. Scale bar, 30 μm.

Finally, to understand how magnetic force interferes with mechanical gating by membrane tension thermodynamically, we analysed the effect of magnetic force application on the kinetics of BBS-2422 channel activation, inactivation, and deactivation elicited by a pressure step (Fig. 5a). We found that magnetic force significantly increases the time constants of activation (4.2±0.3 ms (field off) and 6.1±0.7 ms (field on), *P*<0.01, paired *t*-test) and inactivation (49.0±3.1 ms (field off) and 66.5±6.2 ms (field on), *P*<0.01, paired *t*-test), while leaving deactivation unchanged (11.9±1.5 ms (field off) and 14.6±1.6 ms (field on), *P*>0.01, *n*=19 cells, paired *t*-test; Fig. 5b). These results suggest that the application of a magnetic pulling force not only slows inactivation specifically through a mechanosensitive domain, but also directly inhibits mechanical activation by membrane tension.

## Discussion

We set out to identify domains with high mechanical sensitivity in Piezo1. Current experimental methods do not allow force application on defined sub-molecular structures simultaneous to functional characterization. Here we apply a force of ∼10 pN to specific extracellular domains, while measuring channel function electrophysiologically. Atomic force microscopy experiments have shown that destructive forces that extract an entire transmembrane domain from the lipid bilayer (100–150 pN) or that cause domain unfolding (50–200 pN) are much larger, which is consistent with our finding that none of the tested constructs loses its ability to be mechanically activated under magnetic force application^26^,^27^. Rather, the force in our experiments is comparable to the Coulomb force acting on a single voltage sensor domain of a voltage-gated potassium channel under physiological conditions (∼10 pN; see Methods) or the force acting on a 1 nm long gating spring to open a mechanically activated ion channel (∼10 pN; see Methods)^17^.

Lower forces were indistinguishable from ambient thermal energy and thus did not affect channel behavior. Higher forces could have allowed us to investigate the upper limits of local force application on Piezo1, but the electromagnetic coil in these experiments was already operated near the fusing current. Given this limitation, our measurements of magnetic pulling force on different sized nanoparticles show that the 75 nm diameter size nanoparticles meet a balance by minimizing size while still providing a physically relevant magnitude of force. One related potential caveat in our study arises from the possibility that multiple channels or subunits may be bound to a single nanoparticle. In this case, the force transduced by one nanoparticle would be distributed among these channels, likely rendering the force negligible and therefore resulting in wild-type Piezo1-like behaviour. However, the fact that each channel is maximally labelled to a single nanoparticle is at the same time advantageous, because each labelled channel is then modulated identically.

Our unbiased screen reveals four mechanically sensitive sites in two distinct domains of Piezo1. Sites 86 and 300, located at the proximal N terminus, mark an intriguing location because it resonates with the theoretical mechanism of a gating spring that provides maximal force sensitivity at the outermost end of a lever structure^17^. In addition, an effect in this region also supports structural data suggesting that the numerous transmembrane domains are in fact organized as a ‘blade' structure possibly involved in mechanosensing^4^. Sites 2,422 and 2,425 are part of the outer pore domain, which raises the possibility that mechanisms of inactivation found in other ion channels, such as pore blocking (N-type inactivation) or collapse of the selectivity filter (C-type inactivation), are also at work in Piezos^28^. The fact that single-channel conductance stays unchanged in BBS-2422, which likely interacts directly with the pore region, suggests that it is less likely that the pore helices are being pulled apart by the magnetic force as part of an irreversible structural change. Rather, the lack of an effect on single-channel conductance suggests that we are facilitating a natural movement of the channel by specifically targeting mechanosensitive sites. It is possible, however, that the domains we have identified are critical for channel function but not necessarily its native mechanosensitivity. Therefore we can currently only conclude that these domains are themselves mechanically sensitive, but not necessarily the ‘mechanosensors' of Piezo1.

Despite the N terminus and the outer pore clearly being structurally distinct, it is interesting that these two regions both affect the process of inactivation, though it is still unclear whether this occurs through a common mechanism. A clue for a potential allosteric interaction between these regions may be found in the intracellular ‘beam' structures revealed in the cryo-EM model that span the length of each subunit and through which membrane tension acting on the ‘blade' structures could be transmitted to the pore^4^. In our study, we see that pulling either the N- or C-terminal domain away from the membrane induces a stabilization of the open state on activation by pressure, which manifests itself as a loss of channel inactivation. One possible mechanism by which this occurs could involve a ‘lever and hinge' mechanism by which the ‘blade' domain functions as a stiff lever with its movements allosterically connected to the central pore. Accordingly, our manipulation with a magnetic pulling force may imitate a native tension-induced movement of Piezo1.

Magnetic pulling only affected function of the mechanically activated Piezo1 channel, and not that of the voltage-activated K_v_1.2, even under the added mechanical stress of increased membrane tension. Along with our observation that the effects of magnetic pulling on Piezo1 are confined to only certain domains, this finding suggests that the sites our screen identified in Piezo1 might specifically contribute to sensing mechanical stimulation. Still, it is likely that targeting other specific sites within K_v_1.2 or in entirely different channels could reveal structures not unique to Piezo that can be mechanically perturbed. Our experiments on K_v_1.2 are thus merely a first test for specificity, and future broad application of magnetic pulling could be used to investigate the fundamental principles of mechanical sensitivity in other ion channel proteins.

In light of our findings in Piezo1, it is quite remarkable that we did not observe acute channel activation with the onset of magnetic pulling force, but rather a slowing of activation in BBS-2422. Taken with the idea that a slowing of inactivation indicates a stabilization of the channel's open conformation, this may indicate that perturbing the domains identified in our screen may affect channel activation and inactivation through mechanistically distinct pathways.

Ultimately, we identify two mechanically sensitive domains, and it is possible that more domains remain to be uncovered with more controlled labelling stoichiometry, probing of intracellular sites, or higher forces. This finding sets a direction for future studies in understanding the mechanism of mechanical activation in Piezo ion channels. Working towards a magnetically activated ion channel will be worthwhile beyond understanding Piezo function, as it could provide the means for magnetogenetics—the remote control of neuronal excitability with a magnetic field.

## Methods

### Cloning and characterization

Locations for inserting BBS were selected by identifying poorly conserved residues in *Mus. musculus* (*Mm*) Piezo1 compared to *R. norvegicus*, *H. sapiens*, *M. mulatta*, *C. lupus* and *G. gallus* within predicted extracellular sites. The 13 amino-acid BBS (WRYYESSLEPYPD) or the 10 amino-acid myc-tag (EQKLISEEDL) was cloned into *Mm*Piezo1-IRES-EGFP in the pcDNA3.1(−) vector using the QuikChange II XL Site-Directed Mutagenesis kit (Agilent).

A BBS was cloned into amino-acid position 120 of *Mm*K_ir_2.1 and amino-acid positions 197 (K_v_1.2-BBS-S1-S2), 284 (K_v_1.2-BBS-S3-S4) and 350 (K_v_1.2-BBS-S5-PH) of *H. sapiens* K_v_1.2 in the pcDNA3.1(−) vector. All cloning primers were PAGE purified (Sigma). See Supplementary Table 2 for primer sequences. All clones were fully sequence verified (Genewiz, Inc.) and DNA maxi-prepped for transfection (Promega).

### Cell culture

Human embryonic kidney HEK293T cells (ATCC # 3579061) were provided and authenticated (STR authenticated and verified mycoplasma-free) by the Duke Cell Culture Facility. Cells were cultured at a seeding density of 50,000 cells per well in a 24-well plate in DMEM-HG (Life Technologies) supplemented with 10% heat-inactivated fetal bovine serum (Clontech), 50 U ml^−1^ penicillin and 50 mg ml^−1^ streptomycin (Life Technologies), and grown at 37 °C on Poly-L-lysine and laminin coated coverslips (Sigma). Cells were transiently transfected with *Mm*Piezo1-IRES-EGFP constructs (1.5 μg) in the presence of 10 μM ruthenium red using Fugene6 (Promega) according to manufacturer protocol. The K_ir_2.1-BBS construct was co-transfected with wild-type Piezo1-pIRES-EGFP at a 1:1 molar ratio. The K_v_1.2-BBS construct was co-transfected with EGFP at a 1:0.5 molar ratio. Transfected cells were recorded or immunostained 36–72 h post-transfection.

### Nanoparticle labelling

Nanoparticle labelling process is adapted from a previous study on L-type calcium channels^18^. Transfected cells were washed twice with PBS and incubated for 15 min at 37 °C in PBS containing 1,050 μg ml^−1^ α-bungarotoxin biotin-XX conjugate (1:100; B1196; Molecular Probes) and 10 mM HEPES. Cells were then washed 3 × with PBS for 5 min each, then incubated at room temperature (RT) while rocking gently for 15 min in PBS containing 10 μg ml^−1^ 75 nm streptavidin-coated magnetic nanoparticles (1:100; MHS-075-05; OceanNanoTech) and 10 mM HEPES. Cells were then washed 2 × with PBS and kept at RT during recording for no longer than 1.5 h.

### Immunostaining

Nanoparticle-labelled cells were fixed with 4% paraformaldehyde for 30 min at RT and then blocked with 10% normal goat serum (NGS) for 15 min at RT. Cells were then incubated in 1% NGS containing 1:100 anti-streptavidin antibody (S6390; Sigma) for 1 h at RT, then washed 3 × with PBS and incubated at RT in 1% NGS containing 1:1,000 Alexa Fluor 594-conjugated anti-rabbit antibody (A-11012; Molecular Probes) for 1 hour in the dark. Cells were then washed 2 × in PBS and labelled with 1:100 Alexa Fluor 633-conjugated Wheat Germ Agglutinin (WGA) (W21404; Molecular Probes) for 10 min at RT for membrane visualization. Cells were washed 3 × with PBS, mounted with Fluoromount-G (SouthernBiotech) on glass slides, and imaged on a Zeiss 780 inverted confocal microscope at × 63 magnification. Mean fluorescence intensity was measured along the bounding cell membrane with a custom-written script in Fiji image processing software (see Code Availability).

The custom Fiji script first set a threshold limit on the GFP channel of images to identify GFP positive cells. The threshold processed image was then created into a binary image and filtered with preset Fiji scripts ‘watershed' and ‘erode' to separate and delineate individual cells. Individual objects in the resulting image were then used to define cell shaped ROIs, and the preset Fiji script ‘band' was used to create a 1 μm thick band ROI encircling each cell. Finally, the mean fluorescence intensity was measured within the band for the corresponding immunolabelled nanoparticle channel. The ROIs were visually checked against WGA staining to ensure that they were accurately measuring the cell membrane.

### Double labelling

Efficiency of nanoparticle labelling was tested by first labelling Piezo1-BBS constructs with nanoparticles, and then incubating with 1:100 Alexa Fluor 647-conjugated α-bungarotoxin at a 10 μg/mL concentration in PBS and 10 mM HEPES (B35350; Molecular Probes) at RT for 15 min while rocking. Cells were then washed 3x with PBS for 5 min each, fixed with 4% PFA, and immunostained against streptavidin. For comparison, a separate set of cells were labelled with Alexa Fluor 647-conjugated α-bungarotoxin alone. Membrane fluorescence was measured with the custom Fiji script. Percent labelling efficiency was calculated by:


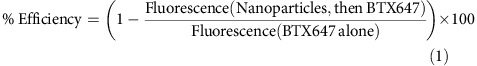


### Myc-tag labelling

Cells transfected with the Piezo1-myc-2422-pIRES-EGFP construct were washed once with pre-warmed culture medium and then incubated in warm medium with 1:50 anti-myc antibody (c-myc 9E11, Santa Cruz Biotech) for 20 min at 37 °C. Cells were then washed 2 × with warm media and 1 × with PBS and kept at RT for recording. For visualization, cells were incubated with 1:200 AlexaFluor546-conjugated anti-mouse antibody (A-11030; Molecular Probes) while rocking gently at RT for 10 min. Cells were then washed 3x in PBS, fixed with 4% PFA, mounted, and imaged with an inverted confocal microscope.

### Electromagnetic needle

The electromagnetic needle was constructed from a HyMU-80 alloy (Carpenter Technology) core (100 mm length, 4.5 mm diameter) and tapered to a ∼10 μM tip with a diamond grinder. The electromagnetic coil was made with *N*=200 turns of enameled AWG 24 copper wire, averaging 5 layers with 40 turns per layer, around a custom made brass frame (30.1 mm length, 7.5 mm inner diameter, 14.5 outer diameter). The copper wire was fixed in place with an epoxy resin coating, and the brass frame with coper wire coil was then sleeved over and clamped onto the HyMU-80 needle 25 mm from the tip of the needle. The electromagnetic coil was then connected to a 12 V DC regulated power supply (301911, Jameco), generating a maximum current of 5 A through the coil. From these parameters, we calculated the magnetic field strength **B** inside the coil to be:


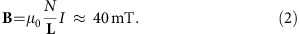


Where *μ* is magnetic permeability, *N* is number of turns, **L** is the length of the coil and *I* is current.

The distance of the needle tip from the patch surface was measured by the displacement readout of the micromanipulator (Sutter Instruments) between the cell surface and the experimental position over several trials.

### Magnetic force calibration

Fluorescent labelling of streptavidin-coated nanoparticles was achieved by first magnetically separating nanoparticles from solution with a permanent magnet (K&J Magnetics) for 5 min and washing once with PBS. Nanoparticles were then blocked with 10% NGS for 15 min while rotating at RT and then magnetically separated again. Next, nanoparticles were resuspended in 1% NGS containing 1:50 anti-streptavidin antibody (S6390; Sigma) and incubated for 1 h while rotating at RT. Nanoparticles were then magnetically separated and washed 3 × with PBS, then resuspended in 1% NGS containing 1:500 AlexaFluor594-conjugated anti-rabbit antibody (A-11012; Molecular Probes). Nanoparticles were magnetically separated and washed 3 × once again before resuspending in PBS. For force measurements, 5 μl of nanoparticle solution was thoroughly mixed with 95 μl of 100% glycerol and placed on a glass coverslip for imaging.

The magnetic force of the electromagnetic needle on 50, 75, 100 and 200 nm diameter magnetic nanoparticles (OceanNanoTech) was estimated by fluorescently labelling and pulling each size nanoparticle separately through 95% glycerol (Sigma), with 5 A of current supplied to the electromagnet, and measuring their velocities within a 50 μm radius from the tip of the needle. For 75 nm nanoparticles, this measurement was repeated with adjusting the supplied current to 1, 2, 3, and 4 A in separate measurements. In this experiment the magnetic force equals the drag force, which was calculated with Stokes law:


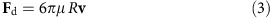


where *μ* the dynamic viscosity, *R* the nanoparticle radius, and **v** the velocity. A force **F**=9.9±0.7 pN was calculated for nanoparticles of 75 nm diameter at 5 A of supplied current.

For comparison, we calculated the Coulomb force **F**_c_ on a voltage sensor domain containing four elementary charges, embedded in a membrane bilayer of 4 nm thickness and exposed to a physiological transmembrane voltage of 60 mV:


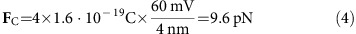


The gating energy (*E*_gating_) transduced by an ideal (Hooke) spring of 1 nm length under a force of 10 pN is substantially larger than the thermal energy (*E*_thermal_) at room temperature (300 K):


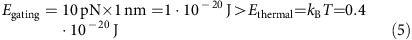


### Electrophysiology

Cell-attached recordings for all experiments were performed at RT in bath solution containing (in mM) 140 KCl, 10 HEPES, 1 MgCl_2_ and 10 Glucose, pH=7.3 with KOH. Pipette buffer for Piezo1 experiments contained (in mM) 130 NaCl, 5 KCl, 10 HEPES, 1 CaCl_2_, 1 MgCl_2_ and 10 TEA-Cl, pH=7.3 with NaOH. Pipette buffer for K_ir_2.1 and K_v_1.2 experiments contained (in mM) 135 NaCl, 5 KCl, 10 HEPES, 2.8 Na-acetate, 1 CaCl_2_ and 1 MgCl_2_, pH=7.3 with NaOH. Patch-clamp recordings were carried out with an EPC10 amplifier and Patchmaster software (HEKA Electronik) and data were sampled at 5 kHz and filtered at 2.9 kHz. Pressure was controlled with a high-speed pressure-clamp system (ALA Scientific Instruments) connected to Patchmaster software. Pipette resistances ranged from 2 to 4 MΩ, and patches were held at −80 mV for the duration of the experiment. Pressure pulses were given at 10 mm Hg steps from 0 to −70 mm Hg for 200 ms, with a +5 mm Hg pulse for 5 s between each step to minimize resting membrane tension^15^. A rest interval of at least 1 min was given between two consecutive pressure-step protocols (that is, before and after magnetic field application). Following an initial pressure-step protocol, the electromagnet was brought within close vicinity (54.7±5.5 μm) directly above the cell-attached patch with a second micromanipulator and was manually switched on for at least 5 s before and for the duration of the second recording.

K_v_1.2 channels were recorded with a voltage-step protocol from −80 mV to +30 mV for 300 ms, separated by a 600 ms period at −80 mV. Within each voltage-step at 100 ms, a negative pressure stimulus of −60 mm Hg was applied for 100 ms, then returned to 0 mm Hg for the remainder of the voltage-step.

### Electrophysiology analysis

Analysis was performed with Igor Pro 6.22A (WaveMetrics). Baseline currents before pressure stimulation were subtracted off-line and peak currents measured at each pressure. The pressure of half-maximal activation (*P*_50_) was calculated by normalizing each set of peak currents to the maximum value for each individual cell and fitting the data with a Boltzmann function:


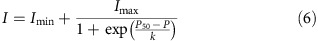


where *I*_min_ and *I*_max_ are the minimum and maximum current amplitude, *P* is pressure and *k* is the slope. The *P*_50_ values for each individual cell were averaged and compared by analysis of variance (ANOVA) and a non-parametric multiple comparison test.

Inactivation was analysed by calculating the normalized difference of the baseline-subtracted maximal current amplitude on a −60 mm Hg pressure-step and the average current amplitude 150 ms later:


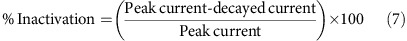


A paired *t*-test was used to determine statistical significance of each construct.

The absolute changes in inactivation without and with the magnetic force were calculated using:


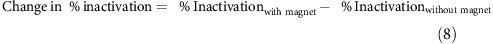


and values were tested for statistical significance by ANOVA followed by Tukey–Kramer comparison.

Time constants for activation *τ*_1_ and inactivation *τ*_2_ were obtained by fitting current I with a double exponential function with bounds after the onset and before the offset of the pressure stimulus:


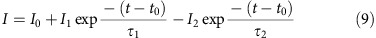


Time constants for deactivation *τ*_3_ were obtained by fitting the current following 40 ms after offset of the pressure stimulus with a single exponential function:


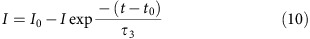


Values of deactivation (*τ*_3_) may have been overestimated due to above threshold oscillations at the offset of the pressure-clamp stimulus. A paired *t*-test was used to determine statistical significance for each construct.

Maximal currents for K_v_1.2 were measured from a P/N leak subtracted voltage-step protocol from −80 to +30 mV for 300 ms divided into 100 ms periods at 0 mmHg, −60 mm Hg, and 0 mm Hg pressure stimulus. A 600 ms period of rest between each step was held at −80 mV. This protocol was repeated once on each cell first without, then with a magnetic field applied. Reversal potentials were extrapolated with a tail current protocol. The calculated conductances for each period and condition were normalized and fit to a Boltzmann function to determine *V*_1/2_ values. Differences in *V*_1/2_ values and normalized maximal conductance were analysed with a paired *t*-test.

### NP_o_ and single-channel analysis

NP_o_ was calculated by measuring the average current amplitude during a 4 s period of the +5 mm Hg prepulse at the onset of each pressure-step protocol and dividing by the maximum current elicited by a saturating −60 mm Hg pressure step.

Single-channel amplitudes were measured by performing a Gaussian multi-peak analysis (IgorPro, Wavemetrics) on histogram plotted current amplitudes over a 1 s period exhibiting single-channel openings. Conductance was then calculated using the holding potential of −80 mV.

### Statistical analysis

For immunostaining experiments, a minimum of *n*=8 cells and two transfections was analyzed for each individual immunostaining study for a maximum s.e.m. of <20% the normalization standard and to account for day to day variability. All analysed electrophysiological recordings had a seal resistance of at least 1 GΩ. Recordings for Piezo1 were only analyzed for patches with maximal pressure-induced currents of at least 40 pA to prevent poor fit. Recordings for K_v_1.2 were only analysed for patches with maximal voltage-induced currents of 50 pA (non-transfected cells, 0/30; wild-type K_v_1.2, 8/15; K_v_1.2 -BBS-S1-S2, 9/24; K_v_1.2 -BBS-S3-S4 8/47; K_v_1.2 -BBS-S5-PH, 0/14). A minimum of *n*=8 (2 transfections) for Piezo1 experiments and *n*=5 cells (3 transfections) for K_v_1.2 experiments were analysed for each individual electrophysiology study for a normal distribution of values for across measurements.

Statistical analysis was performed with paired or unpaired Student's *t*-test and one-way analysis of variance followed by either a non-parametric multiple comparison or Tukey–Kramer comparison. Results are expressed as mean±s.e.m. Significant thresholds were set at *P*<0.01, *P*<0.001, and *P*<0.0001, as defined in the text.

### Code availability

The script for analysing mean near-membrane fluorescence intensity is available in our Github Repository (github.com/GrandlLab).

### Data availability

Source data for (Fig. 2c) showing cryo-EM structures are provided with the article. The authors declare that all other relevant data supporting the findings of this study are available from the corresponding author upon reasonable request.

## 

## Additional information

**How to cite this article**: Wu, J. *et al*. Localized force application reveals mechanically sensitive domains of Piezo1. *Nat. Commun.* **7**, 12939 doi: 10.1038/ncomms12939 (2016).

## Supplementary Material

Supplementary Information Supplementary Figures 1-10, Supplementary Tables 1 & 2

Peer Review File
