# Supplementary material for: Localized force application reveals mechanically sensitive domains of Piezo1
Source: Nat Commun. 2016 Oct 3;7:12939. doi: 10.1038/ncomms12939 (PMC5063965; doi:10.1038/ncomms12939)
Supplement: Supplementary Information — Supplementary Figures 1-10, Supplementary Tables 1 & 2 [file ncomms12939-s1.pdf]

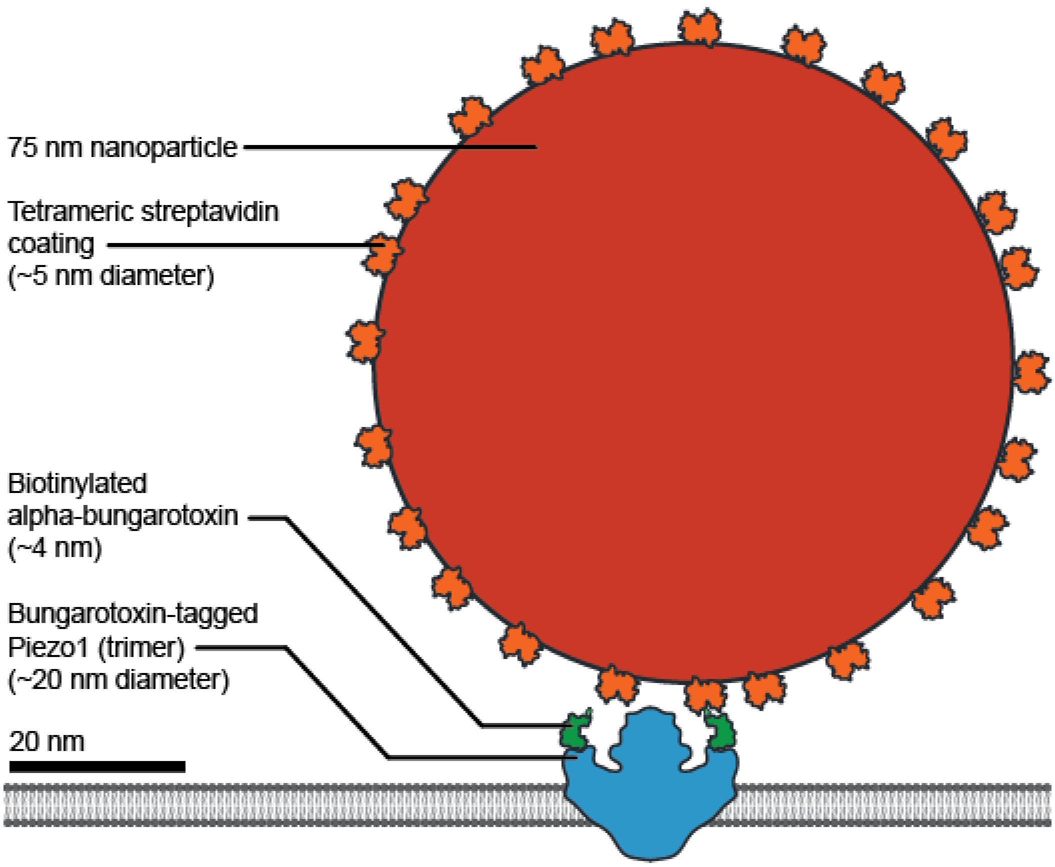

Supplementary Figure 1. Scale diagram of BBS-Piezo1 binding complex. Diagram depicting to-scale relationships of BBS-Piezo1, biotinylated bungarotoxin, and streptavidin-coated nanoparticle in complex.

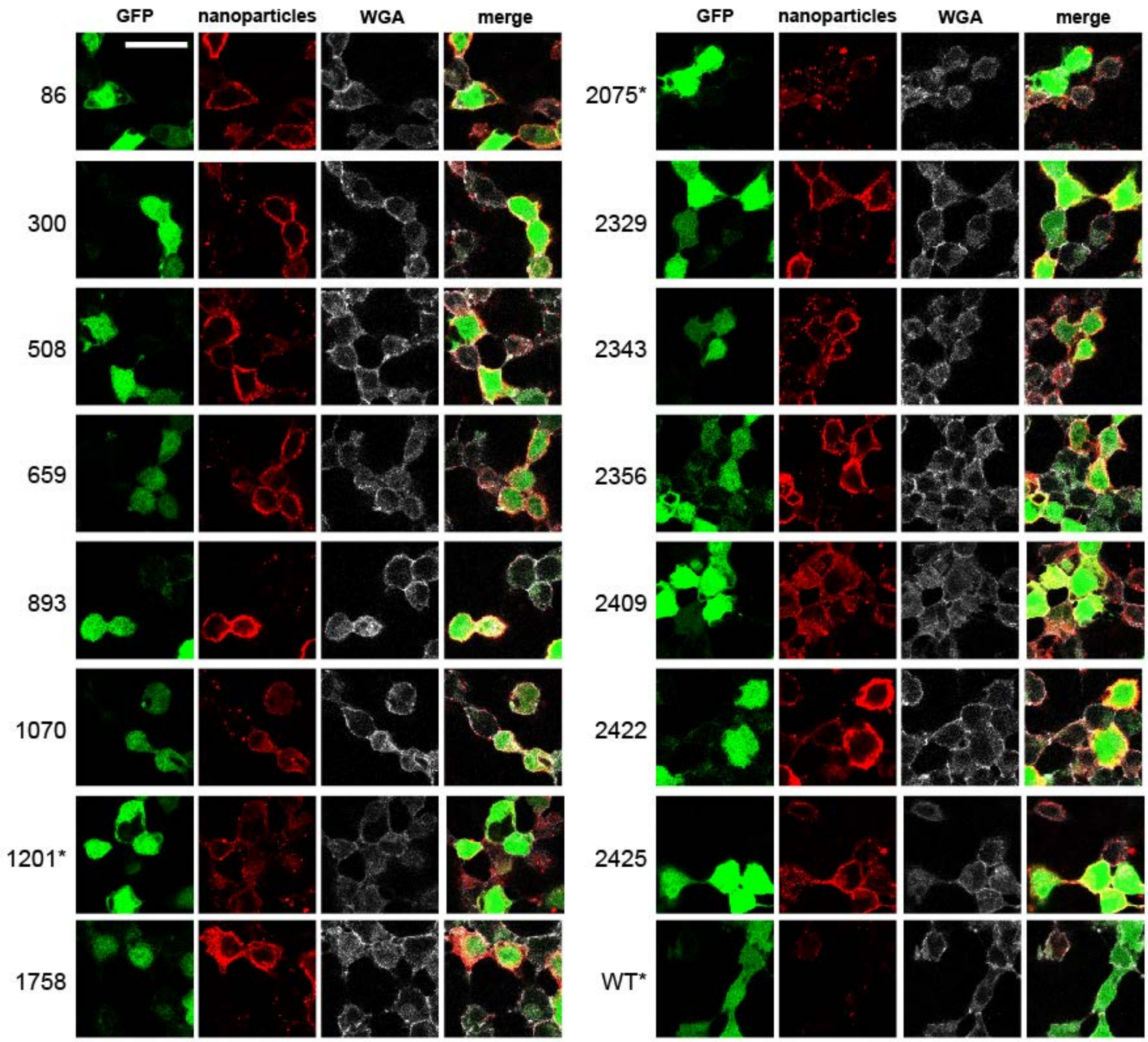

Supplementary Figure 2. Specificity of nanoparticle labeling. Representative images of HEK293T cells expressing Piezo1-BBS constructs, live-labeled with streptavidin-coated nanoparticles, immunostained against streptavidin, and WGA labeled for membrane localization (*, insignificant labeling; green, GFP; red, anti-streptavidin; gray, WGA). Scale bar is 30 μm.

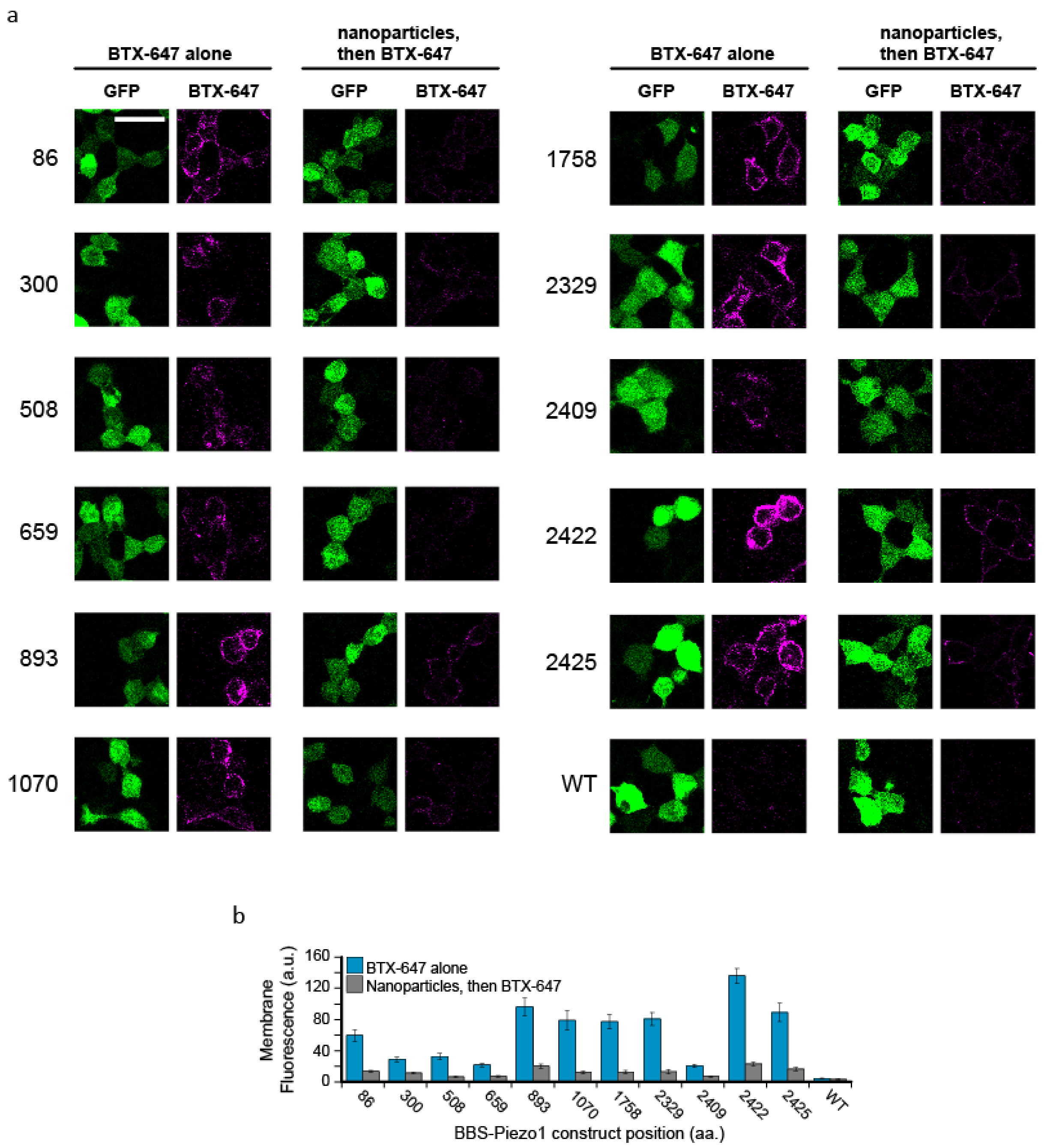

Supplementary Figure 3. Efficiency of nanoparticle labeling. (a.) Representative images of HEK293T cells expressing Piezo1-BBS constructs, live-labeled with either bungarotoxin (BTX)-Alexa Fluor 647 alone or first with nanoparticles, then followed by BTX-647 (green, GFP; magenta, BTX-647). (b.) Membrane fluorescence without (blue) and with (gray) prior nanoparticle labelling. Error bars are SEM. Scale bar is 30 μm.

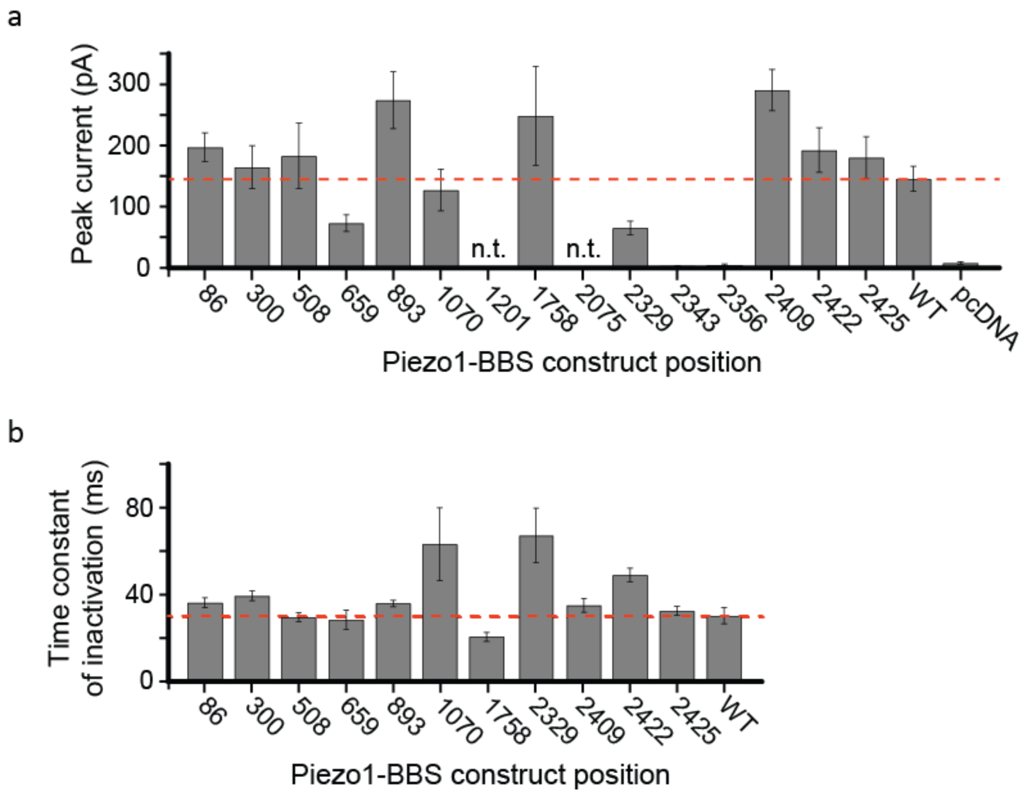

Supplementary Figure 4. Piezo1-BBS construct functionality. (a.) Average maximum current amplitudes during a -60 mmHg pressure step (-110 mmHg step for construct 1758) (n = 8 - 21 cells) (n.t., not tested; BBS-2343 and BBS-2356 significantly lower than WT, $p < 0.0001$, one-way ANOVA and NP multiple comparison) and (b.) Average time constants of inactivation (BBS-1070 and BBS-2329 significantly greater than WT, $p < 0.01$, one-way ANOVA and NP multiple comparison). Red dotted lines denotes averages for wild-type Piezo1. Error bars are SEM.

a

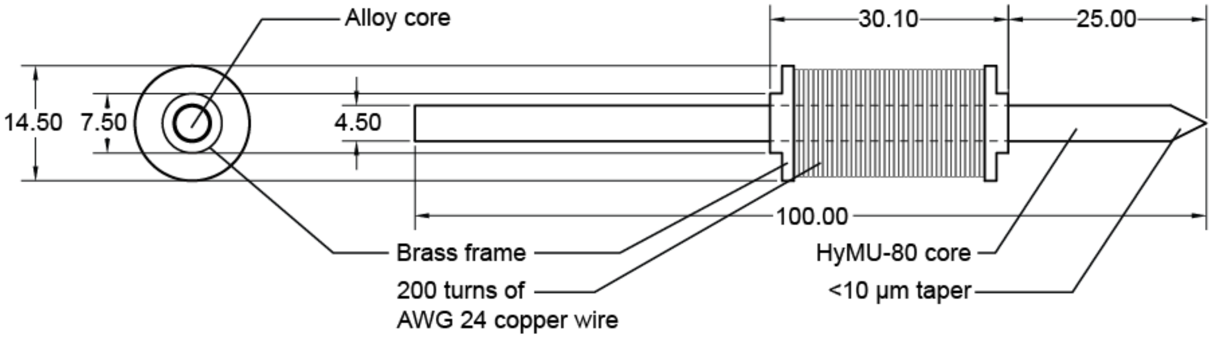

b

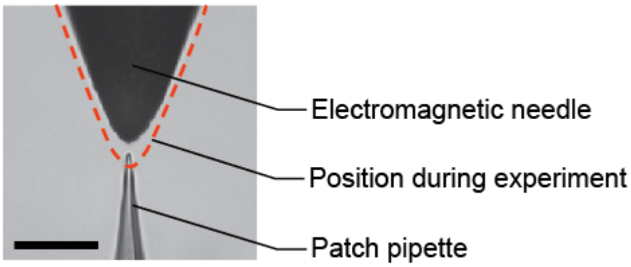

Supplementary Figure 5. Design of electromagnetic needle. (a) Design specifications for electromagnetic needle (units in mm). (b) Image of electromagnetic needle tip and patch pipette tip at 40x magnification. Dotted red line denotes position of needle above pipette tip during recording experiments. Scale bar is 50 μm.

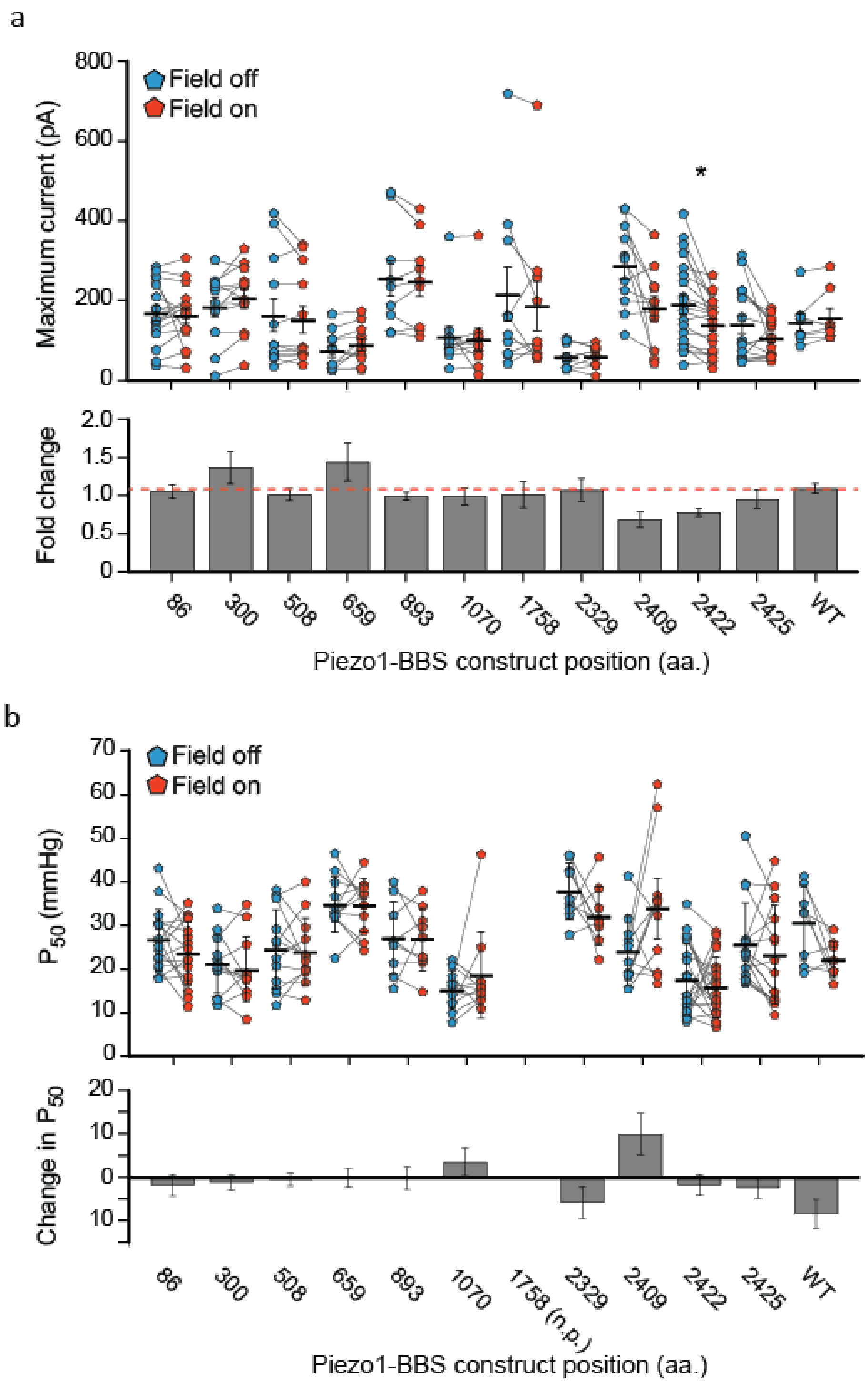

Supplementary Figure 6. Effect of magnetic pulling on BBS-constructs. (a.) Average maximum current amplitudes before and during a magnetic field stimulation (above) (*, $p < 0.01$, paired t-test) and fold change in amplitude (below) ($p > 0.01$ for all constructs, one-way ANOVA and Tukey's comparison) ($n = 8 - 21$ cells) (b.) P50 values before and during magnetic field stimulation (n.p., P50 unavailable for BBS-1758, $p > 0.01$ for all constructs, one-way ANOVA and Tukey's comparison). Error bars are SEM.

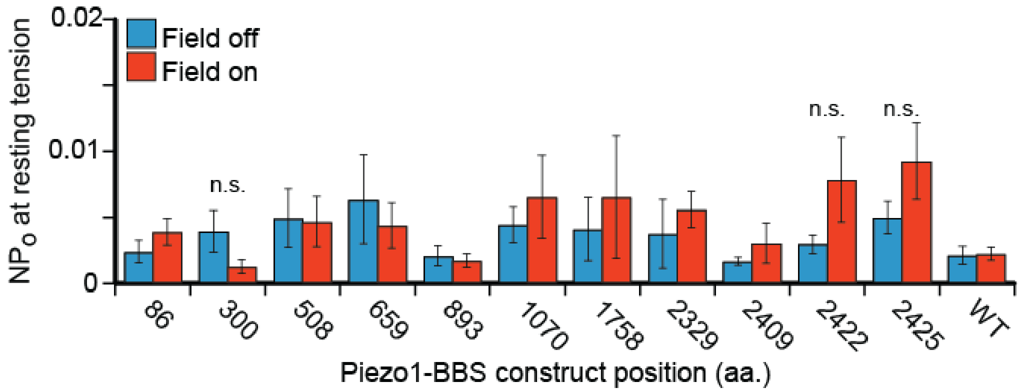

Supplementary Figure 7. Effect of magnetic field on Piezo1-BBS constructs at resting tension. NPo calculated from average current during a 4 second period at resting membrane tension (+5 mmHg) alone or in the presence of a magnetic field (n.s. $p > 0.01$, paired t-test). Error bars are SEM.

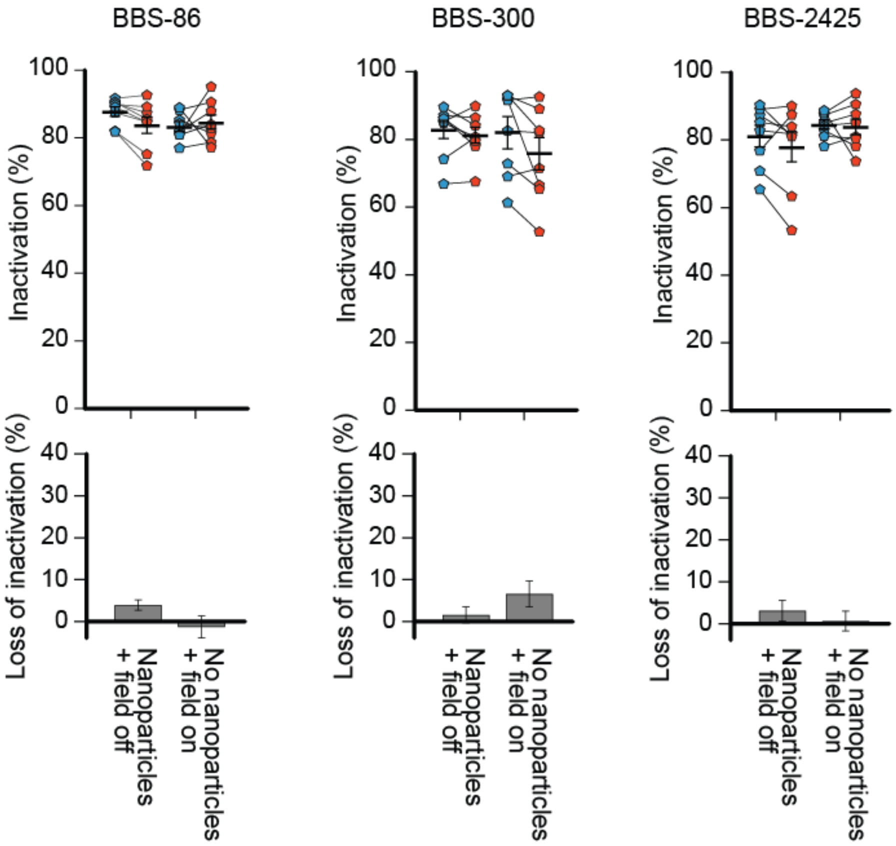

Supplementary Figure 8. Effect of magnetic field and nanoparticle labeling separately. Inactivation of individual constructs BBS-86, BBS-300, and BBS-2425 with nanoparticle labeling alone (magnetic field off) or in the presence of a magnetic field alone (no nanoparticles) ($p > 0.01$ for all comparisons, paired t-test). Error bars are SEM.

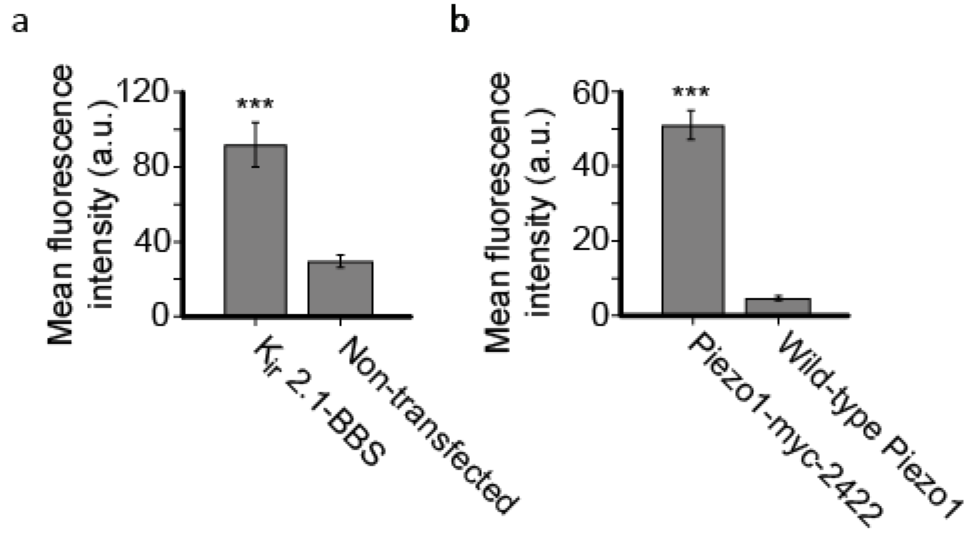

Supplemental Fig. 9. Labeling specificity of tagged constructs. (a) Quantification of mean membrane fluorescence intensity of Kir 2.1-BBS vs non-transfected cells (n = 12 cells each, ***p < 0.0001, unpaired t-test), (b) Piezo1-myc-2422 transfected cells vs wild-type Piezo1 (n = 13 cells each, ***p < 0.0001, unpaired t-test). Error bars are SEM.

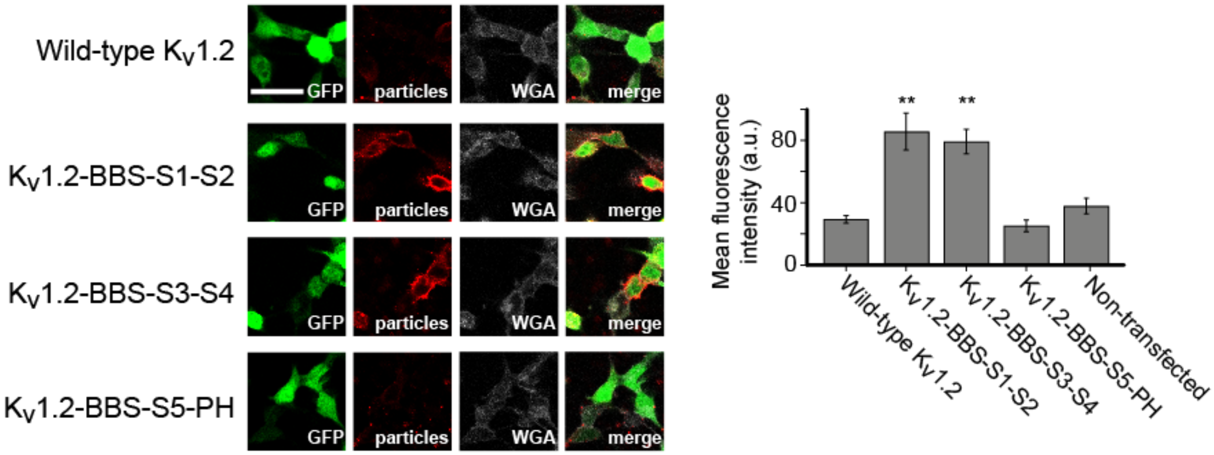

Supplemental Fig. 10. Characterization of K_v1.2-BBS constructs. (a) Representative fluorescent images of HEK293T cells immunostained against nanoparticles for K_v1.2-BBS constructs vs wild-type K_v1.2 (green, GFP; red, nanoparticles; gray, WGA) and quantification of mean fluorescence intensity along cell membrane compared against non-transfected cells (n = 15 cells each, 3 coverslips; **$p < 0.001$, unpaired t-test). Error bars are SEM. Scale bar is 30 μm.

Mean $V_{1/2}$ (mV) for K_v1.2 and K_v1.2-BBS constructs						
	Field off			Field on		
	**A** (0 mmHg)	**B** (-60 mmHg)	**C** (0 mmHg)	**D** (0 mmHg)	**E** (-60 mmHg)	**F** (0 mmHg)
**Wild-type K_v1.2**	-24.4 ± 2.7	-30.4 ± 1.9	-29.3 ± 2.2	-27.4 ± 2.9	-34.3 ± 2.3	-30.5 ± 2.6
**K_v1.2-BBS-S1-S2**	-33.8 ± 2.5	-41.6 ± 3.8	-35.7 ± 2.7	-38.9 ± 3.8	-45.8 ± 4.1	-43.6 ± 4.8
**K_v1.2-BBS-S3-S4**	-39.7 ± 4.9	-44.9 ± 5.5	-41.9 ± 3.8	-42.0 ± 4.7	-46.8 ± 5.3	-43.1 ± 3.4

Supplementary Table 1. **Voltage sensitivity of K_v1.2-BBS constructs**. Mean $V_{1/2}$ values for wild-type K_v1.2 and K_v1.2-BBS constructs ($p > 0.01$ for comparisons between pairs A-D, B-E, and C-F; wild-type K_v1.2, $n = 8$; K_v1.2-BBS-S1-S2, $n = 7$; K_v1.2-BBS-S3-S4, $n = 5$; paired t-test.)

Construct	Forward primers (5' – 3')	Reverse primers (5' – 3')
Piezo1-BBS-86	GCCTACACACCGTGCCTCACTGGAGATACTACGAGAGCTCCCTGGAGCCCTACCCTGACCTGGACCAGTTTCTGGGAC	GTCCCAGAAACTGGTCCAGGTCAGGGTAGGGCTCCAGGGAGCTCTCGTAGTATCTCCAGTGAGGCACGGTGTGTAGGC
Piezo1-BBS-300	TCAAGAACTTCGTAGACCTCTGGAGATACTACGAGAGCTCCCTGGAGCCCTACCCTGACCCTAACTACTCCAGCCCCAA	TTGGGGCTGGAGTAGTTAGGGTCAGGGTAGGGCTCCAGGGAGCTCTCGTAGTATCTCCAGAGGTCTACGAAGTTCTTGA
Piezo1-BBS-508	TGGGCCCTGTCAGCCTGCACTGGAGATACTACGAGAGCTCCCTGGAGCCCTACCCTGACCAGTTGGGACTGGAACACACA	TGTGTGTTCCAGTCCCAACTGGTCAGGGTAGGGCTCCAGGGAGCTCTCGTAGTATCTCCAGTGCAGGCTGACAGGGCCCA
Piezo1-BBS-659	CTTCCAGTTCCAGGACTTCCCCTGGAGATACTACGAGAGCTCCCTGGAGCCCTACCCTGACACCTATTGGCGCAACCTCACG	CGTGAGGTTGCGCCAATAGGTGTCAGGGTAGGGCTCCAGGGAGCTCTCGTAGTATCTCCAGGGGAAGTCCTGGAACTGGAAG
Piezo1-BBS-893	CAACAATACCAACTTGCAGCCTTGGAGATACTACGAGAGCTCCCTGGAGCCCTACCCTGACTTGGAGATCAACCAGTCTTTG	CAAAGACTGGTTGATCTCCAAGTCAGGGTAGGGCTCCAGGGAGCTCTCGTAGTATCTCCAAGGCTGCAAGTTGGTATTGTTG
Piezo1-BBS-1070	TGGCGCTGGAGCAAGGCCATCTGGAGATACTACGAGAGCTCCCTGGAGCCCTACCCTGACCCCATGAATTCCGCCCTCAT	ATGAGGGCGGAATTCATGGGGTCAGGGTAGGGCTCCAGGGAGCTCTCGTAGTATCTCCAGATGGCCTTGCTCCAGCGCCA
Piezo1-BBS-1201	CACTACCCTGCTGCAGAAGTGGAGATACTACGAGAGCTCCCTGGAGCCCTACCCTGACGACACGCGAGCCCAGCTCGTGC	GCACGAGCTGGGCTCGCGTGTCGTCAGGGTAGGGCTCCAGGGAGCTCTCGTAGTATCTCCACTTCTGCAGCAGGGTAGTG
Piezo1-BBS-1758	CCCCTGGAACAGCTACGTTTGGAGATACTACGAGAGCTCCCTGGAGCCCTACCCTGACGTGCTGCGGCGCTATGAGAAC	GTTCTCATAGCGCCGCAGCACGTCAGGGTAGGGCTCCAGGGAGCTCTCGTAGTATCTCCAAACGTAGCTGTTCCAGGGG
Piezo1-BBS-2075	CTGAGAGGATGTTCAGCCAGTGGAGATACTACGAGAGCTCCCTGGAGCCCTACCCTGACAATGCGGTGGCACAGCTGTG	CACAGCTGTGCCACCGCATTGTCAGGGTAGGGCTCCAGGGAGCTCTCGTAGTATCTCCACTGGCTGAACATCCTCTCAG
Piezo1-BBS-2329	CCAAAGGGACCTGGCCAAGTGGAGATACTACGAGAGCTCCCTGGAGCCCTACCCTGACGGTGGCACTGTGGAGTATAC	GTATACTCCACAGTGCCACCGTCAGGGTAGGGCTCCAGGGAGCTCTCGTAGTATCTCCACTTGGCCAGGTCCCTTTGG

Piezo1-BBS-2343	ATGAGAAGCACACCTTGGAGTGGAGATACTACGAGAGCTCCCTGGAGCCCTACCCTGACCTGGCCCCCAACAGTACGGC	GCCGTACTGTTGGGGGCCAGGTCAGGGTAGGGCTCCAGGGAGCTCTCGTAGTATCTCCACTCCAAGGTGTGCTTCTCAT
Piezo1-BBS-2356	GCACGAAGGCAGCTGGCCCAATGGAGATACTACGAGAGCTCCCTGGAGCCCTACCCTGACCTGCTCGAGGGCAGACCTGAC	GTCAGGTCTGCCCTCGAGCAGGTCAGGGTAGGGCTCCAGGGAGCTCTCGTAGTATCTCCATTGGGCCAGCTGCCTTCGTGC
Piezo1-BBS-2409	CAGCTGCGGAGGGAGCAATGGAGATACTACGAGAGCTCCCTGGAGCCCTACCCTGACGTGGGCACAGGGGCCTCTG	CAGAGGCCCCTGTGCCCACGTCAGGGTAGGGCTCCAGGGAGCTCTCGTAGTATCTCCATTGCTCCCTCCGCAGCTG
Piezo1-BBS-2422	GGAGCAAGCGGGCACCAAGTGGAGATACTACGAGAGCTCCCTGGAGCCCTACCCTGACGCCTCCGACTTCCTCGAGTGG	CCACTCGAGGAAGTCGGAGGCGTCAGGGTAGGGCTCCAGGGAGCTCTCGTAGTATCTCCACTTGGTGCCCGCTTGCTCC
Piezo1-BBS-2425	GGCACCAAGGCCTCCGACTGGAGATACTACGAGAGCTCCCTGGAGCCCTACCCTGACTTCCTCGAGTGGTGGGTCATC	GATGACCCACCACTCGAGGAAGTCAGGGTAGGGCTCCAGGGAGCTCTCGTAGTATCTCCAGTCGGAGGCCTTGGTGCC
K_{ir}2.1-BBS	GATACTTCTAAAGTGAGCAAATGGAGATACTACGAGAGCTCCCTGGAGCCCTACCCTGACGCATGCGTGTCGGAGGTCAAC	GTTGACCTCCGACACGCATGCGTCAGGGTAGGGCTCCAGGGAGCTCTCGTAGTATCTCCATTTGCTCACTTTAGAAGTATC
K_v1.2-BBS-S1-S2	GAATGAAGACATGCATGGTTGGAGATACTACGAGAGCTCCCTGGAGCCCTACCCTGACAGTGGGGTGACCTTCCAC	GTGGAAGGTCACCCCACTGTCAGGGTAGGGCTCCAGGGAGCTCTCGTAGTATCTCCAACCATGCATGTCTTCATTC
K_v1.2-BBS-S3-S4	GAGGACGCTCAGCAAGGCTGGAGATACTACGAGAGCTCCCTGGAGCCCTACCCTGACCAGCAGGCCATGTCACTG	CAGTGACATGGCCTGCTGGTCAGGGTAGGGCTCCAGGGAGCTCTCGTAGTATCTCCAGCCTTGCTGAGCGTCCTC
K_v1.2-BBS-S5-PH	GCTGTGTATTTTGCAGAGTGGAGATACTACGAGAGCTCCCTGGAGCCCTACCCTGACGCCGATGAGCGAGAGTCC	GGACTCTCGCTCATCGGCGTCAGGGTAGGGCTCCAGGGAGCTCTCGTAGTATCTCCACTCTGCAAAATACACAGC
Piezo1-myc-2422	GGAGCAAGCGGGCACCAAGGAGCAGAAACTCATCTCTGAAGAGGATCTGGCCTCCGACTTCCTCGAGTGG	CCACTCGAGGAAGTCGGAGGCCAGATCCTCTTCAGAGATGAGTTTCTGCTCCTTGGTGCCCGCTTGCTCC

Supplementary Table 2. **Primer list**. Complementary primer sets used to insert bungarotoxin binding sequence or myc tag (underlined).
