# Supplementary material for: Localized force application reveals mechanically sensitive domains of Piezo1
Source: Nat Commun. 2016 Oct 3;7:12939. doi: 10.1038/ncomms12939 (PMC5063965; doi:10.1038/ncomms12939)
Supplement: Peer Review File [file ncomms12939-s2.pdf]

Reviewer #1 (Remarks to the Author)

The paper explores the important problem of the mechanism of the NhaA antiporter. The focus is placed on so-called constant pH simulations and the results are used to draw mechanistic conclusions. The paper offers a more detailed chain of events in the process of an antiporting cycle, and tries to determine which of two opposing hypothesis regarding pH activation is valid. Also, the paper suggests that K300 rather than D163 is the carrier of the second proton and hints at a structural trigger for the conformational change.
Unfortunately, whereas the paper provides advancement in understanding NhaA's mechanism it suffers from the same problems that prevent most studies in the field from conveying a molecular understanding, namely neglecting the fact that understanding the function of biological systems requires simulating the actual function. Here the authors venture to describe the only consistent simulation study of the function as a non-atomistic study. This issue will be further discussed below, but the problem is in the assumption that a very complex mechanistic action with a time scale of ms can be obtained by MD simulations in the micro- to nanoseconds range. More specifically reference 23 shows how such problems should be studied and what should and should not be modeled with fully atomistic simulations. The pKa's are calculated by what is probabaly at present the most reliable approach (see more below), other parts are modeled by (atomistic) free energy perturbation approaches and then the resulting landscape is used, as it should be, in a time dependent Monte Carlo simulation and provides the first real simulation of the antiporter function.

The present work does not reproduce all the important observed properties of the antiporter (as is the case with all other simulations except ref 23) and have different results in terms of pKa's than those of ref 23 by an approach that is less reliable (see below). After properly describing what is done in ref 23 the present work must discuss the difference in the results considering the points below.
Most of the comments below are given in the order of the corresponding pages:
Page 3 - A major part of the arguments here reflect pKa calculations. Thus, it is problematic to say that PB gave a pKa of 15 without clarifying already in that page that this reflects the incorrect dielectric used in PB approaches and that the PDLD/S-LRA method of ref 23gives probably the most reliable pKa in protein interiors (see "Using a charging coordinate in studies of ionization induced partial unfolding" J . Phys. Chem. B 110, 11566-11570 (2006)). This is the point place to mention the very professional study of pKa results in ref 23. This was not concluded but rather assumed by ref 13.

Page 5 - The findings of ref 4 are discussed, but there is no mentioning that ref 23's model and energy landscapes were able to reproduce and provide an explanation to most of it.

Page 6 - Whereas NapA was shown to be electrogenic, the exact stoichiometry of 2:1 is an assumption (however likely). This should be stated clearly noting that ref 23 reproduced this result.

Page 7 - as stated above, here we need a correct description of ref 23 and not classifying it as non-molecular level.

Page 8 - the implication that so-called constant pH MD is the only way to model the system is really problematic. Not only that the mentioned method does not have correct time scales (since it does not simulate proton transport (which is done in the time dependent MC used in ref 23 where the PMF of each protonation state of the active groups is evlauted with the protonation states of the rest of the groups) but there are alternative ways to study pH dependent conformational changes which include PT (, PROTEINS:Structure, Function, and Bioinformatics 78, 1212-1227 (2010)

Page 8 - here we need a scholarly discussion of the pKa's obtained by ref 23. Furthermore, using constant pH simulations with GB solvent model reflects all the errors of the PB using incorrect

dielectric (read on this issue in 'PROTEINS Structure, Function and Genetics 44, 400-417 (2001).

Page 9 - Can the authors explain why they are using a POPC lipid membrane (which is not very common in E.coli), instead of using the very similar POPE membrane for example?

Page 11 - "Remarkably, as pH increases from 2.5 to 11.5..." the total charge of any given protein is expected to change in the order of N charge units (where N is the number of non-ARG titratable residues) when going from such extreme pH values (which cross all intrinsic pKa's except for ARG). The argument should be made when the pH range is narrower and more relevant to the experiments in the field, i.e. going from ~5.5 to ~9.5 (same for figure 2). Also, the correlation between surface charge and affinity is not well established, references need to be provided .

Page 11 - As is clarified in ref 23 PROPKA is one of the poorest methods for pKa calculations, it works only for surface groups. Also the PROPKA should not be done on the relaxed results from long runs since in this case the site can be stabilized by water molecules (see below).

Page 13 - "Asp164 is the only one among..." there is no closing parenthesis near the end of this sentence.

Page 13 - "consistent with the empirical pKa calculation..." the word empirical is misleading and should be omitted

Page 13 - the correct reference on the problem with GB and PB is the above Protein 2001

Page 14 The results here should be compared to the results of ref 23. Specifially, ref 23 found that the pKa of K300 is higher and that of D163 is lower. Elaboration on the role of the reorientation of the side chains could provide reconciliation.

Page 14 - the way of discussing the observed pH profile is to reproduce it by simulations. This was only done in ref 23.

Page 14 - a pKa of 9 for K300 cannot explain the activity of NhaA at pH 7.5 and similar range (let alone at pH 5 as in ref 4). This has to be discussed.

The results of the constant pH simulations depends critically on the dielectric and when the dielectric is too low the pH induced opening is overestimated - Note that this effect was explored by the overcharging approach J Phys. Chem. B 110, 11566-11570 (2006)).).
Selecting the lysine as the proton acceptor reflects incorrect relaxation for the Asp-Asp interaction that has been explore very carefully by the PDLD/S-LRA which has major atomistic features (e.g. the LRA). The model generated was not shown to even reproduce the pH profile.

No calculations of Na+ binding were given. Ref 23 provides a proper study and also obtained similar results by FEP.

Page 15 - the first computational study to correctly reproduce the pKa in Staphylococcal nuclease is the above JPC 2006 and this should be mentioned. However this does not prove that the present opening is correct since the GB uses unrealistically low dielectric.

Page 19 - the only consistent study of the change of the Na+ profile has been provided in ref 23.

Page 18 - Whereas D164 is readily accessible to the cavity (and therefore to water) D163 is generally buried. It is important to state this to the readers when comparing hydration numbers. Also, to test the suggested connection to the interaction with K300, running the same analysis using a charged D163 but a neutral K300 could be interesting (if not too laborious to perform).

Page 18 - "in agreement with the experimental observation that the activity of NhaA is negligible below pH 6.5..." the introduction itself states that activity was measured at pH as low as 5, please address this.

Page 19 - "while the deprotonation of Asp163 initiates local relaxation..." it is not entirely clear how the previous two paragraphs actually lead to this statement (the second part of the sentence regarding Asp164 is clear)

Page 19 - Is the probability of Na+ binding based on counting frames in which Na+ is bound compared to the length of the simulation? Such an analysis can be dangerous because for example it might lead to overestimating the binding probability due to sporadic sodium trapping within the cavity over a short run (in terms of convergence).

Page 19 - "Interestingly, the pH profile of the ion binding probably for Asp13 nearly coincides with that of the simultaneous..." this statement is rather obvious, since Asp163 is the one that binds 'last' in terms of pH. Considering the narrowness of the cavity at that point and the vicinity of the residues, the curves in figure 5 are highly dependent.

Page 20 - The findings on helix V bending should be put in perspective with the structures of related MjNhaP1 and NapA (which don't seem to exhibit this bending event) and the discrepancy should be discussed.

Page 22 - the fact that the direct simulations cannot reproduce the function is the reason why ref 23 used CG MC approach - this should be discussed clearly.

Page 22 - it is possible that the structural results reflect the low dielectric of the GB.

Page 24 - here again should discuss ref 23's approach since it resolves the problems.

Page 24 - with a proper dielectric the authors might not have found a strong salt bridge.

Page 25 - The authors should discuss at least briefly how their findings fit into Na+ driven transport at constant pH (perhaps at pH 7-8).

.

Page 26 - the only consistent model that emerged from simulations is ref 23 - should be mentioned.

Page 27 - we are told that we have unprecedented level of activation.
However, ref 23 provides much more details and reproduces the full function.

Page 27 - There is no reproduction of a pH profile (specifically, inactivation at high pH) and no mention that ref 23 provided a full explanation to the pH profile. Adding a profile to figure 5 (taking into account proton depletion for example) might be insightful.

Page 27 - it is a problem to see no comparison to relevant experiments.

Page 28 - The PDB ID 1ZCD contains two subunits, whereas the text suggests that it is not the case. Please specify which subunit was used.

Reviewer #2 (Remarks to the Author)

A new computational approach (pH coupled conformational dynamics, CpHMD) has been applied to analyze the mechanism of activity and pH regulation of the Na+/H+ antiporter NhaA. This led to a possible conciliation between the previously suggested allosteric model and the competition model and the new NhaA crystal structure as follows: 1. A pH sensor separated from the active site exists in NhaA (as shown by Padan's group). This is a cluster of residues which collectively respond to a pH signal and attract Na+. It is not allosterically coupled to the active site. 2. The new calculation of pKa of Asp164 and Lys 300 suggests that they are the proton carriers (as suggested by the new crystal structure). 3. Deprotonation of Asp164 opens a cytoplasmic hydrophobic gate and allows for Na+ and water to penetrate the active site. 4. Na+ binds to Asp164 Asp163 and Thr132 in a pH dependent manner. 4. The salt bridge Asp163-Lys300 breaks and the second H+ is released. A clear competition exists between Na and H as suggested by Fendler's group. However as stated above a pH sensor exists. 5. Deprotonation of Lys300 induces bending of TM V which triggers the out ward open conformation.
Taken together the work suggests a new working hypothesis regarding the mechanism of activity and pH regulation of an antiporter and therefore should be accepted.
Major comment. Both the original and new crystal structures show that Lys300 is compensating the partial negative dipoles of the C termini of helixes XIc, XIp. Please discuss it in light of the new pka calculated for Lys300. How the stability of the NhaA fold will be affected?
Minor remarks:
1. Lee et al. showed that NapA is electrogenic, however its Na+/H+ stoichiometry has not been determined. Please change the relevant sentence.
2. This paper does not have any support to the elevator model, I therefore suggest to change the sentence: "The latter triggers a conformational change, possibly
Involving bending of TMV, which may precede a large elevator like conformational transition to the outward-facing state of NhaA" to : "The latter triggers a conformational change, possibly Involving bending of TMV, which may trigger a conformational transition to the outward-facing state of NhaA"

Reviewer #3 (Remarks to the Author)

This is very interesting piece of work, that tries to use very stat of the art simulation methods to study an interesting biological problem. Overall, it is very well done and presented.

The question of which residues are important for Ph dependent mechanisms is interesting in general, and crucial for proton transporters. While experiments can obviously provide many answers, it is really in conjunction with computational studies that a true atomic level details can be found. When speaking about protonation states and the inherent inability of X-rays to see protons, the need to computations is absolutely important.

In this manuscript, the authors use constant pH methods to look at protonation states and conformational changes in NhaA. They show, decisively, that no single residue is to be blamed for the initial sensing events, but rather the whole core acts a single sensor and recruits sodium.

The calculations are done in triplicate, with good convergence data. Control calculations are done to test other hypothesis.

The authors also find that the initial protonation events triggers the opening of a water channels a subsequent conformational change. This is very important finding.

Overall, I find this article worthy of publication in Nature Comms.

If anything, I would ask the authors to suggest some experiment that can verify some of their proposed mechanisms. That was can can figure out if they were right or not, when someone does those experiments.

Reviewer #1

We thank the reviewer for the thorough and critical reading of the manuscript, constructive criticism and detailed comments as well as suggestions. We will address them below.

Comment 1: *Page 3 - A major part of the arguments here reflect pKa calculations. Thus, it is problematic to say that PB gave a pKa of 15 without clarifying already in that page that this reflects the incorrect dielectric used in PB approaches and that the PDLD/S-LRA method of ref 23 gives probably the most reliable pKa in protein interiors (see "Using a charging coordinate in studies of ionization induced partial unfolding" J . Phys. Chem. B 110, 11566-11570 (2006)). This is the point place to mention the very professional study of pKa results in ref 23. This was not concluded but rather assumed by ref 13.*

Response: We agree with the reviewer that the PB calculated pK_a's for internal sites are highly dependent on the assigned effective protein dielectric constant and that the overcharging method provides an efficient way to overcome the energy barrier associated with the ionization-induced conformational change. We added the dielectric constant used in PB calculations on page 3 and a discussion of the calculations in Ref 23 on a later page following the introduction of the new crystal structure which was the basis of the calculations in Ref 23.

Revision: On page 3, bottom:

Based on the first crystal structure of NhaA (6), Poisson-Boltzmann (PB) continuum electrostatics calculations with an effective protein dielectric constant of 4 showed that the pKa's of Asp163 and Asp164 are shifted above 15, ...

On page 6, bottom and continued onto the next page:

Most recently, based on the new crystal structure (20), the function of NhaA was explored using a novel approach which combines the semi-macroscopic simulations for pK_a calculations and Monte-Carlo simulations for evaluating transport models (23). This study supported Asp163 and Asp164 as the two proton carriers (12) and the recent competitive binding (4) and alternating access models (20). Further, it was able to reproduce and rationalize important features of the observed antiport activity, including the 2:1 stoichiometry and pH activity profile (4).

Comment 2: *Page 5 - The findings of ref 4 are discussed, but there is no mentioning that ref 23's model and energy landscapes were able to reproduce and provide an explanation to most of it.*

Response: We added a discussion of the results in Ref 23.

Revision: See revision to comment 1.

Comment 3: *Page 6 - Whereas NapA was shown to be electrogenic, the exact stoichiometry of 2:1 is an assumption (however likely). This should be stated clearly noting that ref 23 reproduced this result.*

Response: We agree with the review and made the revision.

Revision: On page 6, top:

Like NhaA, NapA is electrogenic...

On page 7, top:

Further, it was able to reproduce and rationalize important features of the observed antiport activity, including the 2:1 stoichiometry and pH activity profile (4).

Comment 4: *Page 7 - as stated above, here we need a correct description of ref 23 and not classifying it as non-molecular level.*

Response: We have replaced the previous discussion with the one presented under revision to comment 1. 1–3.

Revision: See revision to comment 1.

Comment 5: *Page 8 - the implication that so-called constant pH MD is the only way to model the system is really problematic. Not only that the mentioned method does not have correct time scales (since it does not simulate proton transport, which is done in the time dependent MC used in ref 23 where the PMF of each protonation state of the active groups is evlauted with the protonation states of the rest of the groups) but there are alternative ways to study pH dependent conformational changes which include PT (PROTEINS:Structure, Function, and Bioinformatics 78, 1212-1227 (2010))*

Response: We agree with the reviewer that constant pH MD is not the only method to model pH-dependent processes and the kinetics of protonation events is not explicitly considered.

Revision: On page 7, second paragraph:

A promising alternative approach that can offer kinetics information has also been developed based on the time-dependent MC simulations and Empirical Valence Bond model (29).

Comment 6: *Page 8 - here we need a scholarly discussion of the pKa's obtained by ref 23. Furthermore, using constant pH simulations with GB solvent model reflects all the errors of the PB using incorrect dielectric (read on this issue in 'PROTEINS Structure, Function and Genetics 44, 400-417 (2001)).*

Response: We added the discussion of the pKa's obtained by ref 23 (see our response and

revision to reviewer's comment 13).

Regarding the systematic errors in PB calculations and CpHMD simulations, please see our response and revision to comment 12.

Comment 7: *Page 9 - Can the authors explain why they are using a POPC lipid membrane (which is not very common in E.coli), instead of using the very similar POPE membrane for example?*

Response: We used POPC lipids to be consistent with the conventional fixed-protonation-state simulations performed by Beckstein and coworker (Lee et al., J. Gen Physiol. 2014). In that study, POPC was used because it was the only lipid type in the OPLS force field that had been extensively validated by long (microsecond) MD simulations. Above being said, to verify if there is a lipid type dependence, additional conventional simulations with CHARMM36 4:1 POPE:POPG mixed membrane were performed (corresponding to S1/S2/S4, 30-470 ns). 4:1 POPE:POPG approximates the composition of the native *E. coli* inner membrane. The same conclusion was obtained, namely, sodium binds in S2: D163(-)/D164(-)/K300(+), and S4: D163(-)/D164(-)/K300(0), but not in S1: D163(-)/D164(0)/K300(+); and the sodium binding sites are T132/D163/D164.

Quantitatively, the binding probabilities are overall somewhat higher in simulations with the native-like mixed membrane, although this may be due to the shorter sampling time as compared to the simulations with POPC. Thus, the conclusions in the current paper remains the same regardless whether POPC or the mixed POPE:POPG native-like membrane is used, consistent with a recent review which found that, unlike ion channels, evidence of lipid dependence for transporters is scant (Denning and Beckstein, Chem Phys Lipids 2013). We added the details of the additional mixed-membrane simulations and conclusions to SI.

Revision: SI, page 2, bottom:

In order to check for a significant dependence on lipids, we performed a second, smaller set of simulations using a 4:1 POPE:POPG membrane which approximates the composition of a native E. coli inner membrane (see Table 1 for further simulation details). The simulations utilized the CHARMM36 protein [22] and CHARMM36 lipid force fields [10], the default CHARMM sodium ion parameters [23] and CHARMM TIP3P water model [6]. The simulations were performed with the same protocol that was previously employed for the simulations of NapA [24].

SI, page 4, table 1 and caption.

**Conventional MD with 4:1 POPE:POPG**[b]			
	PDB	Charge state	Length (ns)
S1	4AU5	D163(-)/D164(0)/K300(+)	30
S2	4AU5	D163(-)/D164(-)/K300(+)	470.5
S4	4AU5	D163(-)/D164(-)/K300(0)	30

[b] These simulations were performed to verify if there is lipid dependence. It was found that sodium binding occurs in S2 and S4 but not in S1. Further, the binding sites are T132/D163/D164. Thus, the conclusions in the main text remain the same regardless whether POPC or a 4:1 POPE:POPG mixed membrane (which approximates the native E. coli inner membrane) is used, consistent with a recent review which found that, unlike ion channels, evidence of lipid dependence for transporters is scant [22].

Comment 8: *Page 11 - "Remarkably, as pH increases from 2.5 to 11.5..." the total charge of any given protein is expected to change in the order of N charge units (where N is the number of non-ARG titratable residues) when going from such extreme pH values (which cross all intrinsic pKa's except for ARG). The argument should be made when the pH range is narrower and more relevant to the experiments in the field, i.e. going from 5.5 to 9.5 (same for figure 2). Also, the correlation*

between surface charge and affinity is not well established, references need to be provided .

Response: On page 11, we revised the relevant sentence. Regarding the pH range, we agree that it is more relevant to use 9.5 as the upper pH in the discussion. However, considering the crystal structure was obtained at pH 3.5, we revised the range to 3.5–9.5. We kept the pH scale in Figure 2 because that was the range used in the simulation.

Regarding the correlation between the net negative charge and ion affinity, we found that it was recognized in the late eighties by Dani through theoretical studies of ion channels (Dani, Biophys. J 1986, 49, pp607). Such correlation was also hypothesized for acid-sensing ion channels that are proton activated and sodium selective (Gonzales et al, Nature 2009, 460, pp599). To reflect this point, we added some discussion.

Revision: on page 9:

Remarkably, as pH increases from 3.5 to 9.5 the net charge decreases from 2.8 to -1, and the sign change occurs around pH 7...

Thus, we suggest that, rather than an individual residue, the pH sensor region collectively responds to the pH signal from the cytoplasm, recruiting sodium and activating NhaA as pH is increased to the active range, consistent with previous observations that the negative potential on the vestibule of ion channels attracts cations into the pore (33, 34).

Comment 9: *Page 11 - As is clarified in ref 23 PROPKA is one of the poorest methods for pKa calculations, it works only for surface groups. Also the PROPKA should not be done on the relaxed results from long runs since in this case the site can be stabilized by water molecules (see below).*

Response and revision: We removed the relevant sentence.

Comment 10: *Page 13 - "Asp164 is the only one among..." there is no closing parenthesis near the end of this sentence.*

Response and revision: We thank the reviewer for catching this typo. We added the missing parenthesis.

Revision: On page 11, top:

Asp164 is the only one among the three acidic core residues that has a pK_a (5.0/6.6/5.8) for the three runs...

Comment 11: *Page 13 - "consistent with the empirical pKa calculation..." the word empirical is misleading and should be omitted*

Response and revision: We deleted the word "empirical".

Revision: On page 11, middle:

...consistent with the PROPKA calculation.

Comment 12: *Page 13 - the correct reference on the problem with GB and PB is the above Protein 2001*

Response and revision: We rewrote the paragraph and added the reference by Schutz and Warshel (ref 37).

Revision: On page 11, bottom and continued onto page 12, top:

The PB-estimated pK_a of Asp164 (about 13) (12) is too high, consistent with the benchmark study of PB-based pK_a predictions of interior residues using the same effective protein dielectric

constant ($\epsilon_\mathrm{p} = 4$) (36). Although overestimation of pK_a shifts can be dampened by adopting a larger ϵ_p to implicitly account for reorganization of the interior polar groups and water penetration, the improvement is limited (36), as a single dielectric constant is insufficient to accurately capture both self energy and charge-charge interactions (37). On the other hand, the CpHMD-estimated pK_a of Asp164 is likely too low (by up to 2 units (38)), as the underlying GBSW model (39) underestimates desolvation (32, 40), due to the use of van der Waals surface which excludes solvent-inaccessible crevices from solute cavity, reflecting the inherent difficulty in treating solute-solvent dielectric transition by GB models (37). However, the extent of the error is fortuitously canceled by the overestimation of desolvation due to inadequate structural relaxation (32, 40) As a result, the calculated pK_a's of internal groups from the hybrid-solvent CpHMD simulations show surprisingly small deviations from experiment (32, 38).

Comment 13: *Page 14 The results here should be compared to the results of ref 23. Specifically, ref 23 found that the pKa of K300 is higher and that of D163 is lower. Elaboration on the role of the reorientation of the side chains could provide reconciliation.*

Response: We compare the calculated pK_a's in Ref 23 and our results based on the same crystal structure (PDB ID 4AU5). In Ref 23, the calculated pK_a of K300 was 12.81, which is 2.81 units higher than our calculated value of 10.1 considering all configurations, with and without sodium binding to D163. However, if separating the sodium-bound and unbound configurations, the CpHMD predicted pK_a's are 8.9 and 11.6, respectively. The latter is higher than the model value of 10.4, in agreement with the value in Ref 23 (12.81). This is within expectation, since in the pK_a calculation of Ref 23, sodium binding was not taken into account (according to our reading of the paper).

Now we compare the calculated pK_a's of D163. In Ref 23, the calculation using implicit charge (assuming neutral background) gave 7.28 with D164(-) and 4.66 with D164(0); the calculation assuming D133(-1)/K300(+) gave 8.4 with D164(-) and 6.5 with D164(0). In our work, the calculated pK_a of D163 was 2.4. This value is not affected by sodium binding, because the latter occurs at a much higher pH (above 6) than the titration range of D163. Since our calculated pK_a's of D133 and D164 were 4.5 and 5.0, both groups were neutral in the titration range of D163. Thus, our calculated pK_a of D163 really corresponds to the result in Ref 23 considering D164(0) and K300(+). Given that Ref 23 gave 4.66 for the pK_a of D163 with D164(0)/K300(0), we anticipate the pK_a of D163 with D164(0)/K300(+) to shift lower (due to electrostatic attraction of K300), bringing it closer to our value of 2.4.

Thus, we think the microscopic pK_a's of K300 and D163 obtained by us and in Ref 23 are in qualitative agreement. To clarify these points, we added discussion in the main text and SI.

Revision: On page 11, end of the first paragraph:

We note that our calculated pK_a of Asp163, which is downshifted from the model value, is in qualitative agreement with the microscopic pK_a obtained by the semi-macroscopic calculations considering neutral Asp164 and charged Lys300 (23) (see SI for detailed comparison). 1 On page 13, bottom and continued onto page 14:

Our calculated pK_a (11.6) in the absence of sodium binding shows an upshift from the model value, which is in qualitative agreement with the pK_a (12.81) obtained by the semi-macroscopic calculations (23) (see SI for detailed comparison),...

SI, page 5:

3. Comparison to semi-macroscopic pK_a calculations

Below we compare the calculated pK_a's of K300 and D163 in Ref. [22] and our results based

on the same crystal structure (PDB ID 4AU5). In Ref. [22], the calculated pK_a of K300 was 12.81, which is 2.81 units higher than our calculated value of 10.1 considering all configurations, with and without sodium binding to D163. However, if separating the sodium-bound and unbound configurations, the CpHMD predicted pK_a's are 8.9 and 11.6, respectively. The latter is higher than the model value of 10.4, in agreement with the value in Ref. [22] (12.81). This is within expectation, since in the pK_a calculation of Ref. [22], sodium binding was not taken into account (according to our reading of the paper).

Now we compare the calculated pK_a's of D163. In Ref. [22], the calculation using implicit charge (assuming neutral background) gave 7.28 with D164(-) and 4.66 with D164(0); the calculation assuming D133(-1)/K300(+) gave 8.4 with D164(-) and 6.5 with D164(0). In our work, the calculated pK_a of D163 was 2.4. This value is not affected by sodium binding, because the latter occurs at a much higher pH (above 6) than the titration range of D163. Since our calculated pK_a's of D133 and D164 were 4.5 and 5.0, both groups were neutral in the titration range of D163. Thus, our calculated pK_a of D163 really corresponds to the result in Ref. [22] considering D164(0) and K300(+). Given that Ref. [22] gave 4.66 for the pK_a of D163 with D164(0)/K300(0), we anticipate the pK_a of D163 with D164(0)/K300(+) to shift lower (due to electrostatic attraction of K300), bringing it closer to our value of 2.4. Thus, we think the microscopic pK_a's of K300 and D163 obtained by us and in Ref. [22] are in qualitative agreement.

Comment 14: *Page 14 - the way of discussing the observed pH profile is to reproduce it by simulations. This was only done in ref 23.*

Response: As the reviewer mentioned earlier, current MD simulations are limited in the time scale. Therefore we did not attempt to calculate sodium flux and compare with experiment. We also agree that time-dependent MC simulations can bridge that gap. We have made revision to reflect this point.

Revision: Page 14, line 6–10:

We note that, due to the limited time-scale of MD simulations, the calculation of sodium flux was not attempted in this work. However, the study by Warshel and coworker (23) was able to provide the pH profile of sodium flux as well as the free energy profile of sodium binding, consistent with experiment (4).

Comment 15: *Page 14 - a pKa of 9 for K300 cannot explain the activity of NhaA at pH 7.5 and similar range (let alone at pH 5 as in ref 4). This has to be discussed.*

Response: We pointed out that the pK_a may be somewhat overestimated due to the fact that the explicit interaction with the sodium ion was not accounted for in the electrostatic calculation for propagating titration coordinates. The discussion was presented on page 13, last part of the first paragraph.

As to pH 5 in ref 4, it refers to the experiment where **saturating** sodium concentration and a pH gradient in the reverse direction (pH 8.5 on peri- and pH 5 on the cyto-plasmic side) were used. Our current simulation can not probe this reverse transport as the conditions are completely different. To clarify this, we made the following revision.

Revision: Page 5, middle:

Electrophysiology experiments showed that, under symmetric, saturating sodium concentration and pH 8.5 on the periplasmic side, reverse transport persists when pH on the cytoplamic side is lowered from 8 to 5 (4).

Comment 16: *The results of the constant pH simulations depends critically on the dielectric and*

when the dielectric is too low the pH induced opening is overestimated - Note that this effect was explored by the overcharging approach J Phys. Chem. B 110, 11566-11570 (2006)).). Selecting the lysine as the proton acceptor reflects incorrect relaxation for the Asp-Asp interaction that has been explore very carefully by the PDLD/S-LRA which has major atomistic features (e.g. the LRA). The model generated was not shown to even reproduce the pH profile.

Response: In the hybrid-solvent CpHMD simulations, conformational dynamics is performed with explicit solvent as in the all-atom MD. In fact, all settings including PME are identical to those in the all-atom MD. GB model is only used to calculate the solvation free energy needed to propagate the titration coordinates, and as such it only affects the pK_a calculation.

That being said, we address below the limitation of GB and the manifestation on the calculated pK_a's.

In the GB framework, the pairwise Coulomb interaction is screened by a distance-dependent dielectric function $f^{\rm GB}$:
$\Delta G^{\rm GB} = -1/2 \sum_{ij} (1 - 1/\epsilon_{\rm wat}) q_i q_j / f^{\rm GB}$, where $f^{\rm GB} = \sqrt{r_{ij}^2 + \alpha_i \alpha_j expt(-r_{ij}^2/F\alpha_i\alpha_j)}$.
From this equations one can see that 1) GB is essentially a "glorified" pairwise Coulomb law, as explained by Warshel and coworker (Schutz and Warshel, Proteins 2011); and 2) The dielectric screening takes place in the function $f^{\rm GB}$, which hides all the complexity, particularly related to the solvent-solute dielectric boundary.

The GBSW model used in this work employs van der Waals surface as the dielectric boundary, which speeds up the calculation but a drawback is that, the small crevices between atoms that are inaccessible to discrete water molecules are treated as solvent, i.e., GBSW model is "too wet". This issue reflects the inherent difficulty in treating the dielectric transition between solute and solvent by GB models, as recognized very early on by Warshel and coworker (Schutz and Warshel, Proteins 2001). As a result, the "effective" dielectric constant for deeply buried sites is too high, which makes the pK_a shifts due to desolvation underestimated. We have observed this systematic error in all of our previous studies employing the same GBSW model, particularly in the 2009 blind pK_a prediction for 87 engineered mutants of Staph nuclease (Wallace et al, Proteins 2011) where we used fully GB-based CpHMD and the subsequent study of the worst prediction case using the hybrid-solvent scheme that eliminates the error due to inaccurate conformational sampling by GB (Shi et al, Biophys J 2012). Note, however, underestimation of desolvation does not occur with GB models based on molecular surface, for example GBMV, although these models suffer from other problems due to the underlying continuum approximation as well as the lack of treatment of the dielectric transition between solvent and solute, as was pointed out Warshel and coworker (Schutz and Warshel, Proteins 2011).

Revision: On page 12, line 3–10:

On the other hand, the CpHMD-estimated pK_a of Asp164 is likely too low (by up to 2 units (38)), as the underlying GBSW model (39) underestimates desolvation (32,40), due to the use of van der Waals surface that excludes the solvent-inaccessible crevices from solute cavity, reflecting the inherent difficulty in treating solute-solvent dielectric transition by GB models (37). However, the extent of the error is fortuitously canceled by the overestimation of desolvation due to inadequate structural relaxation (32,40). As a result, the calculated pK_a's from the hybrid-solvent CpHMD simulations show surprisingly small deviations from experiment for internal groups (32,38).

As to the relaxation of Asp-Asp interaction (assuming the reviewer meant D163 and D164), we performed an additional analysis. It shows that the peak of the distance distribution, which spans 3–8 Å, shifts according to pH and the presence of sodium. The peak shifts to a larger distance, from 5 to 7 Å as Asp163/Asp164 deprotonate at increasing pH. However, when sodium binding

occurs, the peak is shifted back to about 4 Å, reflecting the electrostatic screening due to the ion. Thus, we believe the Asp-Asp interaction is relaxed in the CpHMD simulations.

Revision: In SI, we added a plot (Fig. S20).

Comment 17: *No calculations of Na+ binding were given. Ref 23 provides a proper study and also obtained similar results by FEP.*

Response: We agree that Ref 23 provides a nice binding free energy profile of sodium, which is missing in our study. We added the relevant discussion.

Revision: Page 14, end of the first paragraph:

However, the study by Warshel and coworker (23) was able to provide the pH profile of sodium flux as well as the free energy profile of sodium binding, consistent with experiment (4).

Comment 18: *Page 15 - the first computational study to correctly reproduce the pKa in Staphylococcal nuclease is the above JPC 2006 and this should be mentioned. However this does not prove that the present opening is correct since the GB uses unrealistically low dielectric.*

Response: We thank the reviewer for pointing out the JPC 2006 reference. It was our oversight not to mention it. The reference is now added.

Revision: Page 16, middle:

Opening of hydrophobic cavity and water penetration due to ionization of internal groups has been previously observed in both experimental and computational studies of Staphylococcal nuclease (38,42,43).

As to the potential issue of low dielectric in GB calculations, we addressed it in our response and revision to reviewer's Comment 16.

Comment 19: *Page 19 - the only consistent study of the change of the Na+ profile has been provided in ref 23.*

Response: We agree that the free energy profile of sodium binding obtained in Ref 23 provides valuable insight. Accordingly, among the four possible protonation states for D163 and D164: HH, -H, H-, and –, the last one is most favorable for sodium conduction. This data is in agreement with our finding that the gate opens when both residues are charged. However, what differs is in the "inferred" role of K300. We used the word "inferred" here, because the study in Ref 23 ruled out K300 because of the high pK_a (12.8); however, since a free energy profile for the condition D163(-)/D164(-) with neutral K300 was not provided, we do not really know if such a condition would be even more favorable for sodium conduction as compared to D163(-)/D164(-)/K300(+). Thus, it may well be that our study completely agrees with that of Ref 23. We added a sentence to reflect this point.

Revision: Page 20, end of the first paragraph:

It is also in accord with the recent semi-macroscopic calculations which suggested that sodium binding is most favorable when both Asp163 and Asp164 are charged (23).

Comment 20: *Page 18 - Whereas D164 is readily accessible to the cavity (and therefore to water) D163 is generally buried. It is important to state this to the readers when comparing hydration numbers. Also, to test the suggested connection to the interaction with K300, running the same analysis using a charged D163 but a neutral K300 could be interesting (if not too laborious to perform).*

Response: We thank the reviewer for the insightful comment and helpful suggestion. We added

the information to the main text.

Revision: Page 18, bottom and continued onto the next page:

In contrast, the hydration number of Asp163 remains very low. This is because Asp164 is immediately below the hydrophobic gate readily accessible to the cavity, whereas Asp163 is buried (Fig. 4C) and forms a salt-bridge interaction with Lys300.

Response: As to the second comment, we conducted the additional analysis which confirms that when the hydration number of D163 significantly increases when the D163–K300 salt bridge is broken following the deprotonation of K300. We made the corresponding revision and added the analysis plot to SI.

Revision: We added a plot (Fig. S19) to SI.

We also revised the corresponding sentence in the main text. On page 19, line 4–6:

In contrast, the hydration number of Asp163 sharply increases at pH above 8, which can be attributed to the disruption of the salt bridge with Lys300 following its deprotonation (Supplementary Fig. 19).

Comment 21: *Page 18 - "in agreement with the experimental observation that the activity of NhaA is negligible below pH 6.5..." the introduction itself states that activity was measured at pH as low as 5, please address this.*

Response: We agree the two statements can be made more clear.

The statement on page 18 refers to two experiments: 1) The early experiment (Taglicht et al, J Biol Chem 1991) showed that, under a proton gradient in the forward direction, NhaA becomes activated when the inside (cytoplasmic side) pH is above 6.5. 2) The recent experiment (first part of the study by Mager et al, J Biol Chem 2011) showed that, under a sodium gradient (with concentration below saturation) and symmetric pH, NhaA is active pH above 6.5. Thus, NhaA is active with or without proton gradient as long as the pH is above 6.5, which is supported by our simulations conducted under symmetric pH condition.

The statement in Introduction refers to the recent experiment (second part of the study by Mager et al, J Biol Chem 2011) which showed that, under symmetric, **saturating** sodium concentration and a pH gradient in the reverse direction (pH 8.5 on the outside), reverse transport persists when the inside (cytoplasm) pH is decreased from 8 to as low as 5. This experiment indicates that with high enough sodium concentration, it is possible to activate NhaA even at low proton concentration, thus lending support to the competitive binding mechanism. Our simulations did not have the same conditions as experiments and therefore we did not make direct comparison to this experiment.

To clarify, we revised the corresponding sentences.

Revision: On page 5, second paragraph: Electrophysiology experiments showed that, under symmetric, saturating sodium concentration and pH 8.5 on the periplasmic side, reverse transport persists when pH on the cytoplamic side is lowered from 8 to 5 (4).

On page 18, middle:

Thus, at low pH the cytoplasmic funnel is closed, consistent with experiments showing NhaA is inactive either under a forward proton gradient with an inside pH below 6.5 (3) or under a sodium gradient with symmetric pH below 6.5 (4).

Comment 22: *Page 19 - "while the deprotonation of Asp163 initiates local relaxation..." it is not*

entirely clear how the previous two paragraphs actually lead to this statement (the second part of the sentence regarding Asp164 is clear)

Response: In the analysis of radius of gyration and hydration numbers, we found that upon deprotonation of Asp163, the passage slightly opens (radius gyration increases); however, it is not wide enough for sodium conduction to happen. It was explained on page 17, first paragraph:

“As pH is increased to 4, Asp163 becomes completely deprotonated (charged) and Asp164 remains protonated (neutral); the radius of gyration increases to about 5 Å. Interestingly, the hydration number of Asp164 increases to 2, whereas that of Asp163 remains below 1, and ions remain absent in the core region (later discussion). These data show that, charging of Asp163 leads to slight opening of the gate; however, the passage is not wide enough to accommodate a possibly hydrated sodium ion.”

Comment 23: *Page 19 - Is the probability of Na+ binding based on counting frames in which Na+ is bound compared to the length of the simulation? Such an analysis can be dangerous because for example it might lead to overestimating the binding probability due to sporadic sodium trapping within the cavity over a short run (in terms of convergence).*

Response: We thank the reviewer for the insightful comment. Yes, the probability was calculated by counting frames and as such it could be artificially inflated by the transient binding events. However, we did observe that the residence time is very long. Once a sodium ion is bound, it does not leave in the CpHMD simulations, and in the fixed-protonation-state simulations, the residence time is greater than 400 ns.

Revision: We added a sentence in the caption of Figure 5.

The probability was calculated by counting the number of frames. We note that the residence time of sodium is very long. Once it is bound, it does not leave in the CpHMD simulations. In the fixed-protonation-state simulations, the residence time is greater than 400 ns (20).

Comment 24: *Page 19 - ”Interestingly, the pH profile of the ion binding probably for Asp13 nearly coincides with that of the simultaneous...” this statement is rather obvious, since Asp163 is the one that binds ’last’ in terms of pH. Considering the narrowness of the cavity at that point and the vicinity of the residues, the curves in figure 5 are highly dependent.*

Response: The reviewer is right in that Asp163 is the last to bind and therefore it is a statement of the obvious that its binding coincides with the simultaneous binding to all three residues. We made the following revision.

Revision: On page 19, bottom:

Moreover, Asp163 starts to bind sodium, resulting in the coordination to all three residues, Asp163, Asp164 and Thr132.

Figure 5A: To remove redundancy we deleted the binding probability curve for Asp163.

Comment 25: *Page 20 - The findings on helix V bending should be put in perspective with the structures of related MjNhaP1 and NapA (which don’t seem to exhibit this bending event) and the discrepancy should be discussed.*

Response: We expanded on the discussion.

Revision: On page 27, middle:

Although such movement is not seen in the outward-facing state of NapA (21) and MjNhaP1 from *Methanocaldococcus jannaschii* (49), we hypothesize that, since our simulations only capture the

initial stage of the transport cycle, TM V bending may represent a kinetic intermediate accompanying the large conformational transition to the outward-facing state. Interestingly, it is consistent with the electrophysiology data showing mutation A167P on helix V dramatically slows the rate of conformational transition but does not affect the optimum pH of NhaA (5). To directly verify TM V bending, we suggest to test if the mutation Thr132 to Val132, which eliminates the hydrogen bond with Asp164, would facilitate TM V bending and accelerate the conformational switch.

Comment 26: *Page 22 - the fact that the direct simulations cannot reproduce the function is the reason why ref 23 used CG MC approach - this should be discussed clearly.*

Response: We added a discussion to reflect the powerful utility of the MC based approach.

Revision: On page 14, end of the first paragraph:

However, the study by Warshel and coworker based on MC simulations (23) was able to provide the pH profile of sodium flux as well as the free energy profile of sodium binding, consistent with experiment (4).

Comment 27: *Page 22 - it is possible that the structural results reflect the low dielectric of the GB.*

Response: This issue has been addressed in our response and revision to reviewer's Comment 16.

Comment 28: *Page 24 - here again should discuss ref 23's approach since it resolves the problems.*

Response: We discuss the results from Ref 23 on multiple occasions: page 6, 11, 12, 13-14, 19. In an effort to meet the page limit requirement, we will not duplicate the discussion. Note, the current manuscript is already over the page limit and we have to shorten the text (see Other revision for more details).

Comment 29: *Page 24 - with a proper dielectric the authors might not have found a strong salt bridge.*

Response: In the hybrid-solvent CpHMD simulations, conformational dynamics is performed using explicit solvent as in the conventional MD. In fact, all settings including PME are identical those used in the conventional all-atom MD. GB model is only used for calculation of solvation free energies for propagating the titration coordinates, i.e., the pK_a calculation.

As to the limitation of the GB model and the consequence on the pK_a calculation, please see our response and revision to Comment 16.

Comment 30: *Page 25 - The authors should discuss at least briefly how their findings fit into Na+ driven transport at constant pH (perhaps at pH 7-8).*

Response: We discussed the comparison to that particular experiment in Results.

Revision: On page 18, middle:

Thus, at low pH the cytoplasmic funnel is closed, consistent with experiments showing NhaA is inactive either under a forward proton gradient with an inside pH below 6.5 (3) or under a sodium gradient with symmetric pH below 6.5 (4).

Comment 31: *Page 26 - the only consistent model that emerged from simulations is ref 23 - should be mentioned.*

Response: We provided such discussion in Introduction on page 6.

Revision: On page 6, bottom and continued onto the next page:

Most recently, based on the new crystal structure (20), the function of NhaA was explored by Warshel and coworker using a novel approach which combines the semi-macroscopic pK_a calculations with Monte-Carlo (MC) simulations for evaluating transport models (23). This study supported Asp163 and Asp164 as the two proton carriers (12) and the recent competitive binding (4) and alternating access models (20). Further, it was able to reproduce and rationalize important features of the experimentally observed antiport activity, including the 2:1 stoichiometry and pH activity profile (4).

Comment 32: *Page 27 - we are told that we have unprecedented level of activation. However, ref 23 provides much more details and reproduces the full function.*

Response: We replaced the word "unprecedented level" with "atomic". The discussion of the results from Ref 23 is given in our revision to comment 31.

Revision: On page 26, middle:

Nonetheless, our work reconciles the current models and provides atomic details of the pH-dependent activation and sodium-proton antiport of NhaA.

Comment 33: *Page 27 - There is no reproduction of a pH profile (specifically, inactivation at high pH) and no mention that ref 23 provided a full explanation to the pH profile. Adding a profile to figure 5 (taking into account proton depletion for example) might be insightful.*

Response: This issue has been addressed in our response and revision to reviewer's comment 14. For convenience, we repeat the revision here.

Revision: On page 14, middle:

We note that, due to the limited time-scale of MD simulations, the calculation of sodium flux was not attempted in this work. However, the study by Warshel and coworker based on MC simulations (23) was able to provide the pH profile of sodium flux as well as the free energy profile of sodium binding, consistent with experiment (4).

Comment 34: *Page 27 - it is a problem to see no comparison to relevant experiments.*

Response: We have made comparisons to experiment on multiple occasions in Results. In order to avoid going over the page limit, we will not repeat them here. Note, the main text is already over the page limit. We have to shorten the text in many places (see Other revision at the end of the response letter for details).

Comment 35: *Page 28 - The PDB ID 1ZCD contains two subunits, whereas the text suggests that it is not the case. Please specify which subunit was used.*

Response: We thank the reviewer for catching this oversight. We used subunit A in the structure 1ZCD. This info is now added.

Revision: On Page 28 bottom, continued onto page 29:

CpHMD run 1 was initiated from monomer B of the new crystal structure (PDB ID: 4AU5), while run 2 and 3 were initiated from the subunit A of the previous crystal structure which is a monomer (PDB ID: 1ZCD, same sequence as monomer B in 4AU5).

Reviewer #2

We thank the reviewer for the favorable view of the paper and address the comments below.

Comment 1: *Major comment. Both the original and new crystal structures show that Lys300 is*

compensating the partial negative dipoles of the C termini of helixes XIc, XIp. Please discuss it in light of the new pka calculated for Lys300. How the stability of the NhaA fold will be affected?

Response: We thank the reviewer for the insightful comment. We analyzed the snapshots at different pH conditions using the distance from the amine nitrogen of Lys300 to the backbone carbonyl oxygen of Cys335 (TM XIc) as well as Pro129 (TM IVp). When Lys300 is charged, the interactions between its positive charge and the partial negative dipoles of the C termini of helices XIc and XIp are intact; however, when Lys300 is neutralized, the interactions are disrupted, indicating the destabilization of the NhaA structure. This data suggests Lys300 is charged in the low-pH, inactive structure of NhaA, consistent with the predicted pK_a from CpHMD simulations. We added a short discussion in the main text. The accompanying analysis is added to SI (Fig. S21).

Revision: On page 14, line 3–6:

To further validate the protonation state of Lys300, we examined its interactions with the partial negative dipoles of the C termini of TMs XIc and XIp. When Lys300 is charged, the interactions are intact; however, when Lys300 is neutralized, the interactions are disrupted (Fig. S21). Thus, Lys300 is protonated in the low pH, inactive structure of NhaA.

Comment 2: *Minor remarks: 1. Lee et al. showed that NapA is electrogenic, however its Na+/H+ stoichiometry has not been determined. Please change the relevant sentence.*

Response: We have revised the sentence.

Revision: Like NhaA, NapA is electrogenic and therefore the interaction between Lys300 and Asp163 is likely of functional importance.

Comment 3: *2. This paper does not have any support to the elevator model, I therefore suggest to change the sentence: "The latter triggers a conformational change, possibly involving bending of TMV, which may precede a large elevator like conformational transition to the outward-facing state of NhaA" to : "The latter triggers a conformational change, possibly Involving bending of TMV, which may trigger a conformational transition to the outward-facing state of NhaA"*

Response and revision: We thank the reviewer for the helpful suggestion and we removed the word "elevator-like" from the corresponding sentence.

Reviewer #3

We thank the reviewer for the favorable view of the paper and address the minor comment below.

Comment: *If anything, I would ask the authors to suggest some experiment that can verify some of their proposed mechanisms. That was can can figure out if they were right or not, when someone does those experiments.*

Response: We thank the reviewer for the helpful suggestion. We added the discussion.

On page 27, bottom:

To directly verify TM V bending, we suggest to test if the mutation Thr132 to Val132, which eliminates the hydrogen bond with Asp163, would facilitate TM V bending and accelerate the conformational switch.

Other revision to meet journal formatting requirement:

We meet the formatting requirement of Nature Communications, we made the following changes:

- Abstract has been reduced to 150 words.
- To reduce the number of words in Introduction from nearly 1600 to 1000, we condensed the last three paragraphs into one. We also tightened many sentences.
- To reduce the words in Results and Discussion to 4000, we significantly shortened the section "Agreement between CpHMD, conventional simulations and new crystal structure" and moved the details to SI.
- We also tightened some sentences in Results and Discussion.
- We changed the colors red and green to magenta and cyan, respectively, in all figures.
- We moved the figure legends and table to the end of the document.
